# Factors associated with unmet need for support to maintain independence in later life: a systematic review of quantitative and qualitative evidence

**DOI:** 10.1093/ageing/afac228

**Published:** 2022-10-28

**Authors:** Gemma Frances Spiers, Tafadzwa Patience Kunonga, Daniel Stow, Alex Hall, Andrew Kingston, Oleta Williams, Fiona Beyer, Peter Bower, Dawn Craig, Chris Todd, Barbara Hanratty

**Affiliations:** Population Health Sciences Institute, Newcastle University, Newcastle upon Tyne, UK; Population Health Sciences Institute, Newcastle University, Newcastle upon Tyne, UK; Population Health Sciences Institute, Newcastle University, Newcastle upon Tyne, UK; Faculty of Biology, Medicine and Health, University of Manchester, Manchester, UK; Population Health Sciences Institute, Newcastle University, Newcastle upon Tyne, UK; Population Health Sciences Institute, Newcastle University, Newcastle upon Tyne, UK; Population Health Sciences Institute, Newcastle University, Newcastle upon Tyne, UK; Faculty of Biology, Medicine and Health, University of Manchester, Manchester, UK; Population Health Sciences Institute, Newcastle University, Newcastle upon Tyne, UK; Faculty of Biology, Medicine and Health, University of Manchester, Manchester, UK; Population Health Sciences Institute, Newcastle University, Newcastle upon Tyne, UK

**Keywords:** unmet, review, support, Factors, older people

## Abstract

**Background:**

populations are considered to have an ‘unmet need’ when they could benefit from, but do not get, the necessary support. Policy efforts to achieve equitable access to long-term care require an understanding of patterns of unmet need. A systematic review was conducted to identify factors associated with unmet need for support to maintain independence in later life.

**Methods:**

seven bibliographic databases and four non-bibliographic evidence sources were searched. Quantitative observational studies and qualitative systematic reviews were included if they reported factors associated with unmet need for support to maintain independence in populations aged 50+, in high-income countries. No limits to publication date were imposed. Studies were quality assessed and a narrative synthesis used, supported by forest plots to visualise data.

**Findings:**

forty-three quantitative studies and 10 qualitative systematic reviews were included. Evidence across multiple studies suggests that being male, younger age, living alone, having lower levels of income, poor self-rated health, more functional limitations and greater severity of depression were linked to unmet need. Other factors that were reported in single studies were also identified. In the qualitative reviews, care eligibility criteria, the quality, adequacy and absence of care, and cultural and language barriers were implicated in unmet need.

**Conclusions:**

this review identifies which groups of older people may be most at risk of not accessing the support they need to maintain independence. Ongoing monitoring of unmet need is critical to support policy efforts to achieve equal ageing and equitable access to care.

## Key Points

- Some groups of older people may be more at risk of not getting the day to day support they need.
- These groups include males, the younger old, those living alone and facing greater socioeconomic disadvantage.
- Monitoring which older people have unmet needs is important to support equitable access to care.

## Background

The meaning of independence in later life can take different forms, but broadly reflects having an acceptable degree of autonomy in day-to-day life [1]. Help with essential functional activities like washing, dressing, shopping for groceries, preparing meals and managing medication are critical to supporting older people’s health and independence. Such help may include, for example, a care worker to assist with personal hygiene activities, or dedicated transport services to get to and from places in local communities. People who do not receive the support they need to remain independent are more likely to experience poor quality of life, malnutrition, dehydration, weight loss and falls [2–5]. Lack of help with day-to-day activities is also associated with increased healthcare utilisation [6, 7]. This gap between the need for, and receipt of, support is considered an unmet need. Understanding the patterns of unmet need for support to maintain independence could aid practitioners to deliver care to people who are most in need. Policies to promote equitable access to care services that support independence also require a clear picture of the most underserved populations.

Evidence about factors linked to unmet need for support to stay independent in later life is fast-growing. Yet this evidence is challenging to interpret because the operationalisation of unmet need for support is not standardised. First, there are different ways to identify populations with a need for support: need can be perceived (i.e. people report they need help) or assumed (i.e. people report difficulties staying independent). Second, there are varying ways that a need is considered unmet (Table 1). Absolute measures of unmet need identify those who need, but receive no support. Relative measures of unmet need identify those who need and receive support but who judge such support to be insufficient. A measure of relative unmet need may, therefore, identify a larger population than measures of absolute unmet need. Some studies combine relative and absolute measures, whilst others seek a judgement on perceived unmet need through a direct question, without ascertaining whether a need is unmet because of the absence (absolute) or insufficiency (relative) of support. Third, studies may vary in what type of support they consider relevant to their measure, whether this is paid-for care, unpaid care from family or friends or both.

**Table 1:** Types of unmet need measures

Measure of unmet need	How operationalised
Absolute	From a population with a need for care, identify those who receive no help
Relative	From a population with a need for care, identify those who state they need more help/judge existing help to be insufficient
Combined relative and absolute	From a population with a need for care, identify those who receive no help AND those who need more help/judge existing help to be insufficient
Direct question	A direct question about whether a person perceives themselves to have an unmet need. Unmet need could therefore be absolute or relative, but question may not ascertain whether needs are judged to be unmet because of no help (absolute) or insufficient help (relative).

Each of these variations adds complexity to the evidence about unmet need for support to maintain independence. Perhaps most critical is the differentiation between how need for support is considered unmet. Some authors have noted that absolute measures of unmet need may identify people most in need of care [8, 9]. In contrast, relative measures could be driven by expectations of care, which differ across populations and time [10, 11]. A relative measure may, therefore, under- or over-estimate unmet need, depending on the population. Each approach may identify not only different populations, but also different risk factors for unmet need.

Identifying patterns of unmet need would substantially enhance our understanding of how to support older people’s independence, and have clinical and policy relevance. Clarification about how factors linked to unmet need may or may not differ by the type of measure would also support future investigation. A clear picture of this evidence is long overdue. To address this gap, we aimed to synthesise evidence about factors associated with unmet need for support to maintain independence in later life.

## Methods

A systematic review was conducted to address the study aim (PROSPERO #CRD42021250489). The methods are reported according to PRISMA guidelines below [12].

Two sources of evidence were used as follows:

- quantitative evidence from observational studies, which estimate the association between exposure factors and the outcome unmet need
- qualitative evidence about ageing and support needs to stay independent, which may identify factors not included in the quantitative literature. Preliminary scoping confirmed the qualitative evidence could be identified from systematic reviews of qualitative data, rather than primary qualitative studies.

### Search strategy

A search strategy was developed, piloted and refined (Supplementary Materials are available in *Age and Ageing* online). Evidence sources included bibliographic databases and grey literature (Table 2). Searches were not limited by language, date or publication status.

**Table 2:** Search sources

Bibliographic databases: quantitative studies
MEDLINE (OVID) [1946 to May Week 3], searched 21 May 2021 Embase (OVID) [1974 to 2021 Week 19], searched 21 May 2021 PsycINFO (OVID) [1806 to May Week 3], searched 21 May 2021 HMIC (OVID) [1979 to May 2021], searched 21 May 2021 CINAHL (EBSCO) [1981 to May 2021], searched 21 May 2021
Bibliographic databases: qualitative evidence
MEDLINE (OVID) [1946 to July Week 1], searched 9 July 2021 ASSIA (ProQuest) [1987 to current], searched 12 July 2021 CINAHL (EBSCO) [1981 to July 2021], searched 12 July 2021 EPISTEMONIKOS [to July 2021], searched 12 July 2021
Other sources and grey literature
Reference lists attached to ageing datasets (CFAS, Newcastle 85+, ELSA, the Canadian Longitudinal Study of Ageing, the Health, Ageing and Retirement Study, the Mexican Health and Ageing Study, SHARE, The Irish Longitudinal Study of Ageing, and SWEOLD); Open Grey; Websites that publish potentially relevant literature (NATCEN, NHS Digital, the Health Foundation, and The King’s Fund) Reference lists of included studies

### Review criteria

Studies were included if they reported factors associated with unmet need for support to maintain independence in populations aged 50+ (Table 3). The lower age threshold of 50 years was chosen to capture evidence about risk factors important earlier in the life course. This is important for populations who experience early onset of age-related disability, such as those from lower socioeconomic groups and living in areas of greater deprivation [13–15].

**Table 3:** Review criteria

	Include	Exclude
Population	Populations aged 50+ years. Studies of mixed aged populations will be included if: separate analyses are presented for those aged 50+ years (e.g. through stratification); the average age of the sample exceeds 50 years; or, the majority of the sample are aged over 50 years.	Care home populations.
Exposure	Any factor: Explored in association with unmet need for support to maintain independence (quantitative evidence) Linked to having an unmet need to maintain independence (qualitative evidence).	
Outcome	Unmet need for support to maintain independence (relative, absolute, both, direct question of perceived unmet need). Independence: activities of daily living, instrumental activities of daily living, mobility. Binary, categorical, numerical, or a score-based outcomes of unmet need were eligible.	Studies of prevalence of unmet need, or outcomes of unmet need.
Study design	Observational designs (e.g. cross sectional, longitudinal, retrospective or prospective cohort), or systematic reviews of qualitative evidence; studies published in English using data from high-income countries.	

As the focus of this review was unmet need for support to maintain independence, populations living in residential or nursing care homes were excluded. Populations residing in assisted living or sheltered housing were included.

The outcome was unmet need for support to maintain independence. In quantitative studies, eligible measures of unmet need included absolute, relative, a combination of both, or a question asking if participants perceive themselves to have an unmet need. The need for help may be perceived or assumed. Studies that identified populations with a need for help, but not whether this need was unmet, were not eligible.

Independence was operationalised as functional independence**:** mobility, activities of daily living, instrumental activities of daily living, or social care/long-term care services that support functional independence (e.g. home care, meal services). Studies using measures that combined functional independence needs with other types of need (e.g. health need) were included only if data were presented separately for unmet functional independence needs.

In systematic reviews of qualitative data, eligible reviews were those reporting evidence about perceived unmet need and the factors linked to this. Reviews were ineligible if they reported evidence about need for support to stay independent where it was not clear if that need for support was perceived to be unmet.

No limits were set on the types of exposure factors eligible. For systematic reviews of qualitative evidence, reviews had to report evidence about factors linked to the experience of unmet need for support. Reviews that reported evidence of unmet needs only (i.e. without any evidence of linked factors) were ineligible.

Eligible study designs were observational, including cross-sectional and longitudinal analyses, and systematic reviews of evidence using any qualitative study design (e.g. interview study, ethnography, focus groups). Studies were included if published in English using data from an OECD high-income country [16]. We excluded studies from low- and middle-income countries to enhance comparability of evidence and produce findings relevant to health systems in high-income countries.

### Study selection

Titles and abstracts were screened within Rayyan, an online software platform for systematic reviews [17]. Full texts of selected records were retrieved and assessed against the criteria for inclusion in the review. Publications not available through our own institutions were obtained via the British Library. For both stages of screening, two reviewers screened records independently, and conflicts were resolved through consensus.

### Data extraction, quality assessment and synthesis

Study data were extracted into an Excel spreadsheet by one reviewer and all data checked by a second. Study authors were contacted for clarification where necessary. Quantitative studies were appraised using an adapted version of the Critical Skills Appraisal Programme (CASP) tool for cohort studies [18]. Systematic reviews of qualitative evidence were quality assessed using an adapted version CASP tool for systematic reviews [19]. Quality assessments were undertaken by two reviewers and a final judgement agreed after discussion.

For quantitative studies, a narrative synthesis summarised the direction of the association between the exposure factor and unmet need. The synthesis sought to explore the consistency of associations across studies, rather than quantify summary effects. Where factors were reported across two or more studies, our interpretation also considered whether findings differed by the type of measure of unmet need. To aid the narrative synthesis, data were either visualised in a forest plot or tabulated.

Data were visualised in a forest plot if: two or more studies reported the same factor (exposure), used comparable analytical approaches and reported confidence intervals (or confidence intervals could be calculated). Exposure measures should have been similar enough to allow meaningful judgement of the overall trend in associations across studies. For logistic regressions, coefficients (where reported) were exponentiated into odds ratios for comparability. Where studies used the same measure (e.g. sex) but different referents, data were inverted so that the referent was consistent across studies. Data were plotted using R software [20]. Data not eligible for display on a forest plot were summarised and tabulated.

For the qualitative systematic reviews, data about the identified factors linked to unmet need were tabulated and summarised.

### Integration of quantitative and qualitative evidence

Findings from the quantitative data were grouped into categories. Data from the qualitative reviews were then mapped onto these categories; no additional categories were necessary to accommodate the qualitative data. Findings from the quantitative and qualitative data were then summarised together in each category to identify all factors linked to unmet need across both types of evidence.

## Findings

After screening, 43 primary quantitative studies and 10 systematic reviews of qualitative data were included (Supplementary Materials Table S1a and b are available in *Age and Ageing* online, and Figure 1a and b) [5, 8, 21–71].

**Figure 1:**
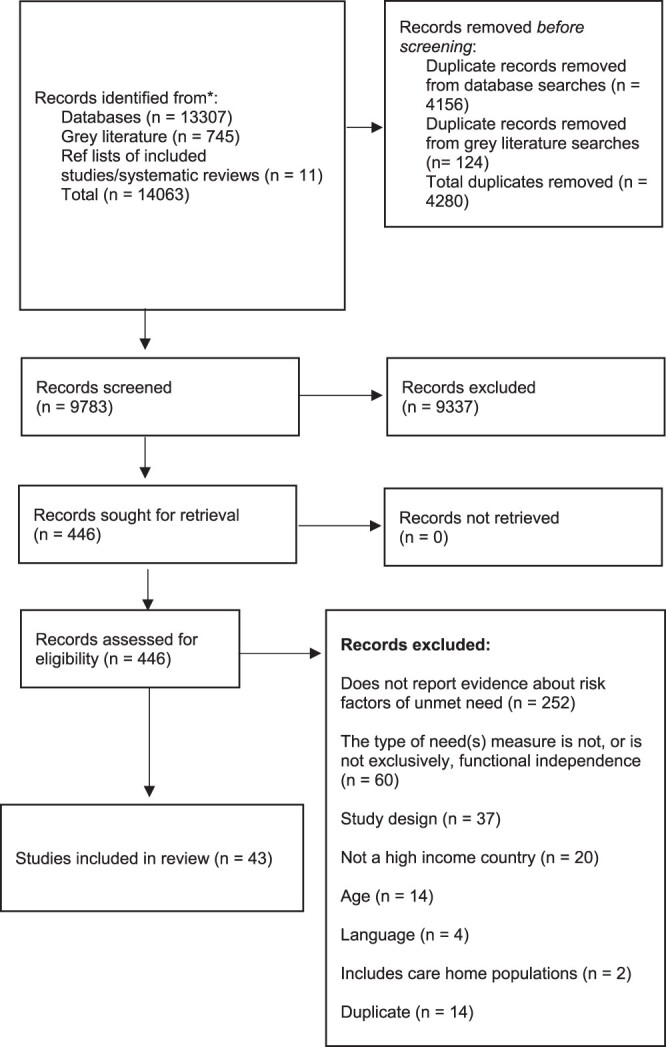
(a) PRISMA Flowchart (quantitative studies). (b) PRISMA Flowchart for qualitative systematic reviews

**Figure 1:**
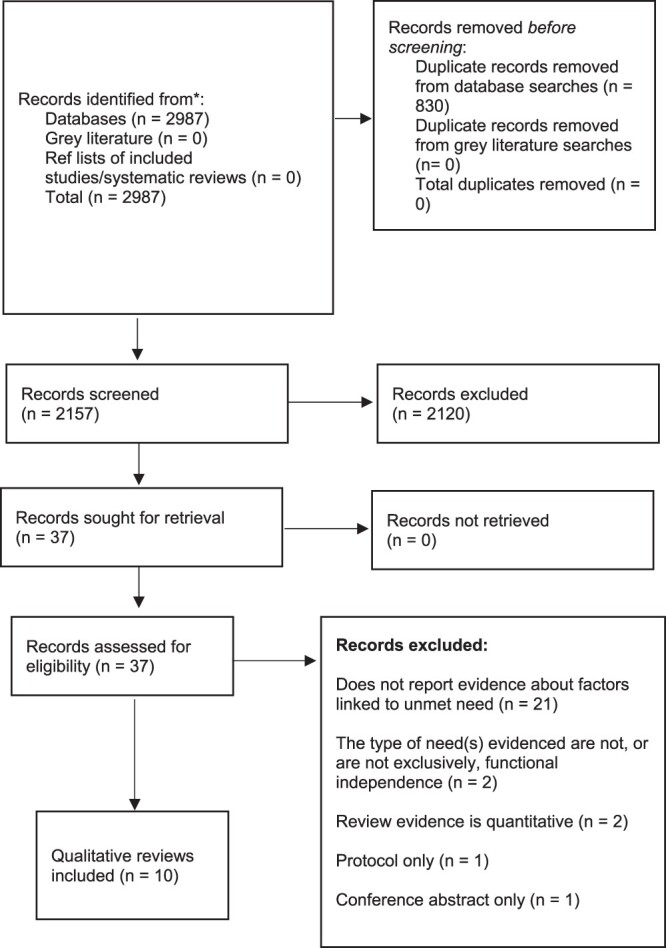
Continued.

The quality assessment assigned major concerns to 12 of the 43 quantitative studies (Supplementary Materials File 2 are available in *Age and Ageing* online). Nine studies were assigned this rating because they did not adjust for any confounding variables. Three were assigned this rating because of potential biases in the representativeness of the sample, missing data and the absence of adjustment for demographic and socioeconomic variables. Given these important methodological limitations, these studies are summarised in Supplementary Materials Table S1 and are available in *Age and Ageing* online, but omitted from the synthesis. No major concerns were identified for the qualitative systematic reviews.

Evidence about seven groups of factors were identified (Table 4). These categories were determined from the data and were not decided a priori. In the following sections, we summarise evidence from each of these groups, integrating quantitative and qualitative data.

### Demographic factors

Supplementary Materials Figure S2a–e, available in *Age and Ageing* online, summarise the evidence about sex, age, marital status, living arrangements and ethnicity. A pattern of evidence suggested that being male, younger age groups, living alone, and black, Hispanic and ‘other’ ethnic populations were linked to unmet need.

Other demographic factors linked to unmet need included being a carer, living in a household of more than 3 people, and living with children (Supplementary Materials Table S2 are available in *Age and Ageing* online). In the qualitative reviews, demographic factors linked to unmet need included: living alone, proximity of friends/family, and cultural and language barriers.

### Socioeconomic factors

Supplementary Materials Figure S3a–e, available in *Age and Ageing* online, summarise the evidence about education, occupation, income, housing tenure and Medicaid insurance status. Categories of higher incomes (compared to categories of lower income) were linked with a lower odds of unmet need in most studies reporting this factor. There was no clear pattern of evidence about education, occupation, housing tenure or Medicaid status (a programme of health insurance in the USA for low income populations). Other socioeconomic factors linked to a greater odds of unmet needs included having a mortgage (compared to those who owned their home outright), a low/medium standard of living, and fair/poor housing quality, and lower non-housing wealth (Supplementary Materials Table S3 are available in *Age and Ageing* online). In the qualitative reviews, socioeconomic factors linked to unmet need included perceived financial constraints.

**Table 4:** Groups of evidence about factors linked to unmet need

Type of factors	Quantitative evidence	Qualitative evidence
Demographics	✓	✓
Socioeconomics	✓	✓
Health/disability	✓	
Health service use	✓	
Care configurations	✓	✓
Unpaid carer characteristics	✓	
Area level measures	✓	

### Health and disability factors

Supplementary Materials Figure S4a–j, available in *Age and Ageing* online, summarise the evidence about self-rated health, presence of functional difficulties, number or volume of functional difficulties, physical functioning score, presence of a limiting illness, types of health conditions and the number of health conditions. In most studies, good or excellent self-rated health was associated with lower odds of unmet need compared to fair or poor self-rated health. Evidence also linked presence of functional difficulty and a greater number of functional difficulties, to a greater odds of unmet need.

Supplementary Materials Figure S4g–o, available in *Age and Ageing* online, shows data for individual health conditions reported across two or more studies. There was some evidence that a greater number of depression symptoms and greater depression severity were linked to unmet need. Other health factors linked to unmet need were arthritis, pain and constipation (Supplementary Materials Table S4 are available in *Age and Ageing* online).

### Health service use factors

Health service use data are summarised in Supplementary Materials Table S5 and are available in *Age and Ageing* online. Reporting a dental visit was associated with a greater odds of unmet needs (1 study).

### Care configuration factors

Care configuration data are summarised in Supplementary Materials Table S6 are available in *Age and Ageing* online. Greater levels of unmet need were reported by care recipients who received 10+ hours of unpaid care a week compared to those receiving less than this amount. A greater volume of paid and unpaid care combined was linked to lower risk of unmet need. Evidence about other care configuration factors and unmet need were inconclusive. From the reviews of qualitative evidence, care factors linked to unmet need included: the quality and adequacy of care, absence of services, eligibility criteria, changes to care staff, a reluctance to burden family and refusing or not seeking help.

### Unpaid carer factors

Supplementary Materials Figure S5a–d, available in *Age and Ageing* online, summarises data about carer sex, age, educational attainment and self-rated health. There was no clear trend of evidence about unmet need and carers’ age, sex and educational attainment. Compared to very good or excellent carer health, fair, poor or bad carer health were associated with a greater odds of the care recipients’ unmet needs. Other factors linked to unmet need were longer durations of caring (in years) and providing care across a greater number of care domains (Supplementary Materials Table S7 are available in *Age and Ageing* online).

### Area level factors

One study reported evidence about long-term care coverage and unmet needs in the US (Supplementary Materials Table S8 are available in *Age and Ageing* online). Populations aged 85+ had higher probability of unmet need than those aged under 85 years in states with higher rates of populations in care homes.

### Type of measure

Overall, there was no strong evidence to suggest that the type of unmet need measure shaped the pattern of findings for factors. The exception was sex: most studies that did not use absolute measures demonstrated that men were less likely to have unmet need. Of studies that used absolute measures, most showed men were more likely to have unmet needs.

## Discussion

The gap between the need for, and receipt of, care in older populations is taking on greater importance in both policy and practice. Although global estimates of the size of this gap are limited, we know that in the UK, around 1.4 million older people who experience difficulties with ADLS have needs that go unmet [72]. Understanding which groups of older people are most likely to have unmet needs is a critical step in efforts to ensure every older person gets the help necessary as they age. Our review has addressed this and identified factors linked to unmet need for support to maintain independence in later life.

The majority of evidence described demographic, socioeconomic and health and disability factors. For factors reported across multiple studies, being male, younger, living alone, lower income, poor self-rated health, greater severity and number of depression symptoms, and more functional limitations were linked to unmet need. Although not all studies reported statistically significant results, the overall pattern in the direction of associations suggest these factors are likely to be important. Evidence from the qualitative reviews confirmed the importance of living alone and perceived financial constraints in unmet need, which align to the quantified evidence on these factors. To some extent, these findings also fit with what is known about the barriers faced by older people when accessing health services. For example, evidence shows that poorer ageing populations are less likely to access health care [73]. Similarly, older people living alone with disability are more likely to experience delayed access to health services [74].

The role of other factors, including those relating to the receipt of paid and unpaid care, sources of care and carer characteristics, was unclear. Other evidence from the qualitative reviews implicated aspects of care (e.g. eligibility criteria, the quality, adequacy and absence of care) and cultural and language barriers in unmet need.

Our synthesis also considered the type of unmet need measure—absolute, relative, absolute and relative combined, or a direct question. Overall, the type of measure did not appear to drive the pattern of results. Only evidence for sex hinted that findings may differ by measure. This may reflect potential differences between men and women about expectations of paid and/or unpaid care, although evidence is needed to ascertain this.

### Strengths and limitations

To our knowledge, this is the first systematic review of factors associated with unmet need for support to maintain independence in later life. With no publication date limit, and the inclusion of quantitative and qualitative data, our work offers a comprehensive and up-to-date picture of this vast and complex evidence.

There is no single approach to defining unmet need. Within the context of social care, studies take different approaches to: identifying need, defining how it is unmet, and the types of support (paid or unpaid). We adopted an operationalisation of unmet need that accommodated these different approaches. This enabled a synthesis that maximised the inclusion of useful evidence, but inevitably increased heterogeneity in the findings.

An inclusive view of need for care goes beyond functional independence. Meaningful participation within society and connectedness with others are also critical social care needs [75]. However, studies typically defined (unmet) need in terms of a person’s mobility and their ability to carry out basic and instrumental activities of daily living. This most likely reflects the data available within cohort studies. We therefore acknowledge that our conclusions are limited to unmet need for support that relates to functional independence only.

A measured interpretation of the finding about younger age and unmet need should consider that categories of age groups varied across studies. We therefore use the term ‘younger’ to make a relative comparison to the oldest age categories. This means that we are unable to infer from our synthesis which ages are most likely to be linked to unmet need.

Finally, the extent to which these factors represent independent effects, and the potential for factors to moderate others, is unclear. Therefore, some factors may be more important than others in driving unmet need. Further work could explore this and identify which risk factors could be prioritised when targeting support.

### Implications for policy and practice

Globally, equitable access to long-term care is a policy priority [76]. Efforts to achieve this could target older people whose needs are more likely to go unmet. Contextual factors implicated in unmet need must also be addressed, including the availability, quality and adequacy of care. Governments considering reforms to long-term care funding and eligibility should pay close attention to the link between socioeconomic disadvantage and unmet need. This aligns closely with what is already known about the link between disadvantage and access to wider healthcare.

Policy makers advocating approaches that prevent and postpone later-life dependency may wish to consider the finding that younger age is linked to unmet need. This finding may reflect emerging needs that are not yet considered eligible or severe enough for intervention. However, support needs that are not adequately addressed at younger ages could potentially lead to a more detrimental loss of independence earlier in the life course. Targeting support as early as possible is therefore critical.

Finally, regular monitoring of which groups are least likely to access the support they need will support policy efforts to enhance equitable access to care. The adoption of unmet need metrics is therefore a critical consideration for future cohort studies of ageing, as well as administrative sources of data collection within health and long-term care.

## Conclusion

Unmet need for support to maintain independence is an important indicator of access to care. This review identifies which characteristics of older people which may increase their risk of not accessing the support needed to maintain independence. Ongoing monitoring of unmet need is critical to support policy efforts to ensure older people are supported when needed.

## Supplementary Material

aa-22-0904-File002_afac228
