# Supplementary material for: Factors associated with unmet need for support to maintain independence in later life: a systematic review of quantitative and qualitative evidence
Source: Age Ageing. 2022 Oct 28;51(10):afac228. doi: 10.1093/ageing/afac228 (PMC9618284; doi:10.1093/ageing/afac228)

**Factors associated with unmet need for support to maintain independence in later life: a mixed methods systematic review (SUPPLEMENTARY MATERIALS)**

Contents

[MEDLINE Search Strategies 2](#_Toc112051112)

[Summary of included studies 4](#_Toc112051113)

[Data summary tables 13](#_Toc112051114)

[Figures 29](#_Toc112051115)

# MEDLINE Search Strategies

**Quantitative studies**

| 1. | Care poverty.ab,kw,ti. |
| --- | --- |
| 2. | "Health Services Needs and Demand"/ |
| 3. | "Health Services Accessibility"/ |
| 4. | Needs Assessment/ |
| 5. | ((Unmet or insufficient or unfulfilled) adj3 (need* or care or demand* or requirement* or support)).ab,kw,ti. |
| 6. | (need* adj3 (care or caring or support*)).ab,kw,ti. |
| 7. | or/1-6 |
| 8. | "Activities of Daily Living"/ |
| 9. | "Disabled Persons"/ |
| 10. | ((Activit* or independen*) adj3 living).ab,kw,ti. |
| 11. | (BADL or BADLs or PADL or PADLs or IADL or IADLs or ADL or ADLs).ab,kw,ti. |
| 12. | (independen* adj3 (loss or lost)).ab,kw,ti. |
| 13. | ((dependen* or disab*) adj3 (care or caring or support*)).ab,kw,ti. |
| 14. | (onset adj2 disabilit*3).ab,kw,ti. |
| 15. | or/8-14 |
| 16. | exp aged/ or middle aged/ |
| 17. | 7 and 15 and 16 |

**Systematic reviews**

| 1. Independent living/ |
| --- |
| 2. ((Unmet or insufficient or unfulfilled) adj3 (need* or care or demand* or requirement* or support)).ab,kw,ti. |
| 3. (need* adj3 (care or caring or support*)).ab,kw,ti. |
| 4. ((Activit* or independen*) adj3 living).ab,kw,ti. |
| 5. physical abilit*.ab,kw,ti. |
| 6. mobilit*.ab,kw,ti. |
| 7. (BADL or BADLs or PADL or PADLs or IADL or IADLs or ADL or ADLs).ab,kw,ti. |
| 8. (independen* adj3 (loss or lost)).ab,kw,ti. |
| 9. "living alone".ab,kw,ti. |
| 10. ((dependen* or disab*) adj3 (care or caring or support*)).ab,kw,ti. |
| 11. ((aging or ageing) adj2 (experienc* or place or perspective*)).ab,kw,ti. |
| 12. (onset adj3 disabilit*).ab,kw,ti. |
| 13. or/1-12 |
| 14. ((primary or social or home or community) adj2 (care or services)).ab,kw,ti. |
| 15. (elder* adj2 care).ab,kw,ti. |
| 16. or/14-15 |
| 17. Older people.ab,kw,ti. |
| 18. (exp aged/ or middle aged/ or "Aged, 80 and over"/) not child/ |
| 19. or/17-18 |
| 20. Qualitative research/ |
| 21. (qualitative or ethnography or scoping or systematic or interview).ab,kw,ti. |
| 22. or/20-21 |
| 23. meta-analysis.pt. |
| 24. meta-analysis/ or systematic review/ or meta-analysis as topic/ or "meta analysis (topic)"/ or "systematic review (topic)"/ or exp technology assessment, biomedical/ |
| 25. ((systematic* adj3 (review* or overview* or analys*)) or (methodologic* adj3 (review* or overview* or analys*))).ti,ab,kf,kw. |
| 26. (((quantitative or narrative) adj3 (review* or overview* or synthes*)) or (research adj3 (integrati* or overview*))).ti,ab,kf,kw. |
| 27. ((integrative adj3 (review* or overview*)) or (collaborative adj3 (review* or overview*)) or (pool* adj3 analy*)).ti,ab,kf,kw. |
| 28. (data synthes* or data extraction* or data abstraction*).ti,ab,kf,kw. |
| 29. (handsearch* or hand search*).ti,ab,kf,kw. |
| 30. (mantel haenszel or peto or der simonian or dersimonian or fixed effect* or latin square*).ti,ab,kf,kw. |
| 31. (met analy* or metanaly* or technology assessment* or HTA or HTAs or technology overview* or technology appraisal*).ti,ab,kf,kw. |
| 32. (meta regression* or metaregression*).ti,ab,kf,kw. |
| 33. (meta-analy* or metaanaly* or systematic review* or biomedical technology assessment* or bio-medical technology assessment*).mp,hw. |
| 34. (medline or cochrane or pubmed or medlars or embase or cinahl).ti,ab,hw. |
| 35. (cochrane or (health adj2 technology assessment) or evidence report).jw. |
| 36. (comparative adj3 (efficacy or effectiveness)).ti,ab,kf,kw. |
| 37. (outcomes research or relative effectiveness).ti,ab,kf,kw. |
| 38. ((indirect or indirect treatment or mixed-treatment) adj comparison*).ti,ab,kf,kw. |
| 39. ((meta-narrative or meta-ethnograph* or mixed method* or critical or thematic or realist or framework or scoping or systematic) adj3 (review* or synthes*)).ti. |
| 40. or/23-39 |
| 41. (13 or 16) and 19 and 22 and 39 |
| 42. A new conceptual model of experiences of aging in place in the United States.m_titl. |
| 43. unmet care needs of older people.m_titl. |
| 44. (Identifying and understanding the health and social care needs of older adults with multiple chronic conditions and their caregivers).m_titl. |
| 45. Experiences of health care for older people who need support to live at home.m_titl. |
| 46. (Older persons' experiences and perspectives of receiving social care).m_titl. |
| 47. (Understanding the care and support needs of older people).m_titl. |
| 48. or/42-47 |
| 49. 41 and 48 |

# Summary of included studies

Table S1a. Summary of included quantitative studies

| **Study author and date** | **Country** | **Data source** | **Population: generic or diagnostic group** | **Population: age  Mean/median (SD/IQR) OR range OR lower age threshold id** | **Sample size** | **Measure of unmet need** | **Types of factors** | **Analysis** |
| --- | --- | --- | --- | --- | --- | --- | --- | --- |
| Adam 1999 | US and UK | Aging and Health Dynamics in the Oldest Old survey and the GB General Household Survey | Generic | 70+ | United States (n = 1,847) Great Britain (n = 1,203) | Absolute | Demographics, care configurations, health/disability. Health service use | Logistic Regression |
| Allen 1994* | US | Patient survey | Cancer | Males: 63.9(10.1) Females: 55.3(11.1)  Range: 28-84 | 353 | Both absolute and relative | Demographics, socioeconomic status, care configurations, health service use, caregiver characteristics. | Logistic Regression |
| Agree 1999 | US | Survey of Asset and Health Dynamics of the Oldest Old | Generic | 70+ | 1,509 | Potentially both^a^ | Demographics, socioeconomic status, care configurations, health/disability | Ordinal regression |
| Amjad 2016* | US | National Health and Aging Trends Study | Dementia | 65+ | 7609 | Unclear^b^ | Health/disability | Logistic Regression |
| Andrew 2014 | Australia | Australian Stroke Clinical Registry and National Stroke Foundation | Post stroke | 68 (IQR: 59,77) | 521 | Relative | Demographics, health/disability | Logistic Regression |
| Beach 2016 | US | National Health and Aging Trends Study and National Study of Caregiving | Generic | 65+ | Caregivers (n = 1,996) Care receivers (n = 1,366) | Absolute | Demographics, health/disability, care configurations | Logistic Regression |
| Berridge 2018 | US | Medicare enrolees | Generic | 65+ | 6,459 | Absolute | Demographics, socioeconomic status, health/disability, health service use | Logistic Regression |
| Branch 1981* | US | Framingham Cohort Study | Generic | 55+ | 2634 | Direct question of perceived need | Demographics | Chi-square |
| Brimblecombe 2017 | UK | Online survey of public sector employees in England who provide unpaid care | Generic | 60.1% aged 65+ | 150 | Direct question of perceived need | Demographics, care configurations, health/disability, caregiver characteristics | Logistic Regression |
| Burchardt 2018 | UK | UK Family Resources Survey | Generic | No data on age but separate analyses reported for 65+ | 15,133 (>65 sample only) | Absolute | Demographics, socioeconomic status, health/disability | Regression |
| Carriere 2000 | Canada | Health and Activity Limitation Survey | Generic | 65+ | 35797 | Relative | Demographics | Descriptive cross-tabs |
| Casado 2012 | US | Participants recruited from various sources^c^ | Generic | 78.6 (9.2) | 146 | Absolute | Care configurations, health/disability, health service use, caregiver characteristics | Logistic Regression |
| Casado 2011 | US | National Long-Term Care Survey | Generic | 65+ | 1021 | Absolute | Demographics, care configurations, health/disability, caregiver characteristics | Logistic Regression |
| Chen 1998* | Canada | Health and Activity Limitation Survey of | Generic | 65+ | 2907 | Both absolute and relative | Demographics, socioeconomic status | Descriptive cross-tabs |
| Davey 2013 | US | wave of the National Long-Term Care Survey | Generic | 60+ | 2,422 | Absolute | Demographics, socioeconomic status, health/disability, area factors | Logistic Regression (nested models) |
| Desai 2000 | US | National Health Interview Survey’s Supplement on Aging | Generic | 70+ | 1346 | Relative | Demographics, socioconomic status, health/disability | Logistic Regression |
| Dunatchnik 2016 | UK | Waves 1 and 6, English Longitudinal Study of Ageing | Generic | 65+ | 749 | Both absolute and relative | Demographics, socioeconomic status, health/disability | Logistic Regression |
| Edward 2020* | US | Health and Retirement Survey | Cognitive Impairment | 55+ | 4666 | Absolute | Health/disability | Descriptive cross-tabs |
| Gousia 2021 | UK | All waves of English Longitudinal Study of Ageing | Generic | 69.0 (9.84) | 81,328 | Both absolute and relative | Demographics, socioeconomic status, health/disability | Logistic Regression |
| Hermsen 2018 | Netherlands | Participants recruited from 22 general practices (GPs) in the region of Amsterdam | Joint pain | 76.8 (6.3) | 407 | Both absolute and relative | Care configurations, health/disability | Logistic Regression |
| Hlebec 2015 | Slovenia | SHARE, Slovenian respondents only | Generic | 74.5 (No SD reported) | 1,372 | Absolute | Demographics, socioeconomic status, health/disability | Logistic Regression |
| Jackson 1991* | US | National Long-Term Care Survey | Generic | 65+ |  | Absolute | Demographics, socioeconomic status, health/disability | Descriptive cross-tabs |
| Kemper 2007 | US | Medicare Current Beneficiaries Survey | Generic | 78.0 (no SD) | 6067 | Absolute | Demographic, socioeconomic status, area factor. | Logistic regression |
| Kennedy 2001 | US | National Health Interview Surveys | Generic | 55.3 (no SD) | 5050 | Both absolute and relative | Demographic, socioeconomic status, care configurations, health/disability | Logistic Regression |
| Kuzuya 2008* | Japan | Nagoya Longitudinal Study for Frail Elderly | Generic | No difficulty: 78.9 (6.8) Difficulty + assistance: 81.4 (8.0) Difficulty + no assistance: 79.9 (7.6) | 1875 | Absolute | Demographic, socioeconomic status, care configurations, health/disability, health service use | Chi-square |
| La Plant 2004 | US | National Health Interview Survey | Generic | 61.4 (23.1) | 9646 | Both absolute and relative | Demographics, socioeconomic status, health/disability | Regression |
| Li 2006 | US | National Long-term care survey | Generic | 79.5 (7.5) | 265 | Relative | Demographic, socioeconomic status, care configurations, health/disability | Logistic Regression |
| Li 2012* | US | Participants recruited from numerous sources^d^ | Alzheimer’s Disease | 66.6 13.6 | 109 | Both absolute and relative | Demographics, care configurations, health/disability, health service use | Regression |
| Liem 2004 | Canada | Administrative patient data | Spinal Cord Injury | Needing more help/unmet need: 9.3 (10.7) Not needing more help/met need: 56.3 (10.1) | 352 | Relative | Demographics, health/disability | Logistic Regression |
| Marcheselli 2018* | UK | Health survey for England | Generic | 65+ | 8,178 | Absolute | Demographics, socioeconomic status, health/disability | Descriptive cross-tabs |
| Otero 2003 | Spain | Baseline data Ageing in Leganes Study | Generic | 76.0 (7.6) | 1135 | Both absolute and relative | Demographic, socioeconomic status, care configurations, health/disability | Logistic Regression |
| Potter 2019 | US | National Health and Aging Trends study | Generic | 65+ | 3694 | Both absolute and relative | Demographic, socioeconomic status, care configurations, health/disability | Logistic Regression |
| Schure 2015 | US | Native Elder Care Study | Generic | 55+ | 505 | Both absolute and relative | Demographic, socioeconomic status, health/disability | Logistic Regression |
| Siegal 1991 | US | Participants recruited from outpatient clinics | Cancer | Mean: 60 (SD: 12.5) | 483 | Both absolute and relative | Health/disability, caregiver characteristics | Logistic Regression |
| Shea 2003 | Sweden, US | US: Medicare Current Beneficiary Survey  Sweden: Ageing at Home Survey | Generic | 75+ | US sample: 4583 Swedish sample: 1378 | Absolute | Demographics, socioeconomic status | Logistic Regression |
| Tennstedt 1995 | US | Geographically stratified sample from 19 localities in Massachusetts | Generic | 70+ | 235 | Absolute | (For community sample only): Demographic, care configurations, health/disability | Logistic Regression |
| Van der Roest 2009* | Netherlands | Participants recruited from various sources^e^ | Dementia | 79.8 (7.6) | 236 people with dementia 322 informal carers | Both absolute and relative | Caregiver characteristics | Chi-square |
| Vlanchantoni 2019 | UK | English Longitudinal Study of Ageing | Generic | 65+ | Unmet ADL weighted: 1103 Unmet IADL weighted: 1107 Unmet mobility weighted: 2191 | Absolute | Demographic, socioeconomic status, health/disability | Logistic Regression |
| Wilkinson-Meyers 2013 | New Zealand | Participants recruited from 60 practices in three District Health Board regions | Generic | 75+ | 3057 | Both absolute and relative | Demographic, socioeconomic status, health/disability | Logistic Regression |
| Willink 2019* | US | National Health and Aging Trends Study | Generic | 65+ | 7070 | Absolute | Demographics, socioeconomic status | Descriptive cross-tabs |
| Yang 2018* | US | National Social Life, Health and Aging Project | Generic | 70.9 (0.3) | 1703 | Absolute | Health/disability | Chi-square |
| Zhou 2018 | Chile | Survey of National Dependency of Older Adults | Dementia | 72.2 (8.5) | 445 | Both absolute and relative | Demographics, socioeconomic status, care configurations, health/disability | Negative binomial regression |
| Zuverink 2020 | US | Round 1 to Round 6 of the National Health and Aging Trends Study | Generic | 65+ | 3936 | Absolute | Demographic, socioeconomic status, care configurations, health/disability, health service use | Cox proportional hazards |

*Assigned major concerns in quality rating and thus not included in synthesis; ^a^Respondents were asked if they have difficulty performing six activities of daily living (eating, dressing, bathing, inside mobility, transferring and toileting) regardless of assistance received. Therefore, it is not clear if participants had no assistance (absolute) or some assistance (relative), or both; ^b^Unmet needs operationalised as a lack of help, but it is not clear if this refers to no help (absolute) or insufficient help (relative); ^c^Sources: county agencies for the aging, social service agencies and organizations, senior centers, and churches that serve Korean Americans in the area;^d^Sources: referrals to a treatment center for AD, local outpatient clinics, and caregiver support groups sponsored by the local Alzheimer’s Association; ^e^Sources: the Centrum Indicatiestelling Zorg, two memory clinics, ten meeting centers for people with dementia and their informal carers and three psychogeriatric day care centres;

Table S1b. Study summary and findings from systematic reviews of qualitative evidence

| **Study author and date** | **Country** | **Population** | **Number of studies in review (date range)** | **Findings: Type of need** | **Findings: How need judged to be unmet** | **Findings: Factor linked to unmet need** | **Does review meet 4/5 DARE Criteria?** |
| --- | --- | --- | --- | --- | --- | --- | --- |
| Abdi 2019 | UK | Chronic conditions. Living in the community.  65+ | 40 (2008 - 2018) | Home support, self-care, domestic activities, mobility, problems in social and community life. | Authors state that 'frequent unmet needs were also reported in areas where informal carers were the main source of support' (p.8)  A need for more/increased access to care. | **Informal support inadequate or unreliable  Living alone**  **Proximity of family/friends** | Yes |
| de Sao Jose 2016 | Portugal | Generic, living in the community.  65+ | 30 (1990 - 2012) | Home care/social care not otherwise specified. | Needs and preferences were overlooked. | **Being excluded from care decisions by care workers** | Yes |
| Dostalova 2021 | Czech Rep | Generic, older adults living in own homes. 58-102 years. | 15 (13 unique studies) (2009 - 2015) | Physical restrictions such as reduced mobility, loss of physical capability, visual and hearing impairments; personal hygiene care, household activities, food preparation, medication management | Older people received no help or inadequate help through carers neglecting older person's needs or not completing work. | **Insufficient time available for care**  **Care workers having negative attitudes, neglecting needs, showing insufficient interest in client’s needs.** | Yes |
| Fjordside 2016 | Denmark | Generic, older adults living in own homes. No age thresholds given. | 12 (11 unique studies), (2011 - 2014) | Home care, support to live independently. | Inadequate help. Feeling neglected and overlooked by carers. | **Carers unresponsive to needs**  **Not involving care recipient in decisions about their care**  **Time-limited care**  **Absence of required services** | Yes |
| Gregory 2017 | Australia | Older people who need support to live at home.  60+ | 46 (1998 - 2015) | Post-discharge home support. | Access to needed support was inhibited. | **Staffing changes**  **Lack of knowledge about services**   **Lack of provider empathy limiting quality of care** | Yes |
| Johnson 2018 | Canada | Older people receiving home care.  65+ | 50 (2000 - 2016) | Home care not otherwise specified. | Review findings include the theme 'Unmet needs' with subthemes relating to home care, but no elaboration is provided on how needs judged to be unmet. | **Eligibility criteria** | Yes |
| Kwan 2019 | England | Older people receiving home care.  60+ | 17 (2010 - 2013) | Personal care and support to live independently at home. | Inadequate help. Authors state "older people said that lack of interest in, or respect for, their views left them feeling that their care was not designed to meet their personal needs" and "older people’s changing needs were not often recognised" (p.13). | **Care workers lack of interest in/respect for recipients’ views leaving care needs unmet.**   **Time available from carers**  **Language barriers** | Yes |
| Lommi 2015 | Italy | Older people living in own home, cognitively unimpaired.  65-96 years. | 11 (1998 - 2013) | Functional limitations, staying independent. | Refusing help that was otherwise judged to be required. | **Refusing or not seeking help** | Yes |
| McGilton 2018 | Canada | Adults with multiple chronic conditions.  55+ | 45 (2003 - 2017) | Home care, activities of daily living, maintaining lifestyles. | A lack of coordinated home care.  Authors also state that 'They [older people] also articulated the need to maintain current capacity to perform activities of daily living but for many, maintaining lifestyles was difficult' (p.27) | **Disagreements in perceived level of support needed**    **Absence of home care services** | Yes |
| Rosenwohl-Mack 2020 | US | Older people living in the community.  No age threshold. | 38 (37 unique studies) (1998-2018) | Ageing in place, remaining in the community. | Authors describe 'a significant gap between [older people's] ideals or preferences for aging in place and the reality of their lived experiences.' (p.17)  Also: 'those who did accept care but were not able to access sufficient or appropriate support reported unmet needs' (p.17) | **Financial constraints**  **Discrimination in care**  **Lack of information about services**  **Cultural or language barriers to care**  **Reluctance to burden family members**   **Poor and insufficient care** | Yes |

# Data summary tables

Table S2 Demographic data not included in forest plots

| **Study** | **Outcome** | **Exposure (factor)^a^** | **Estimate** | **Lower CI** | **Upper CI** | **P** |
| --- | --- | --- | --- | --- | --- | --- |
| Adam 1999 | Unmet ADL need (OR) | Great Britain (ref: United States) | 1.02 | 0.83 | 1.25 |  |
| Agree 1999* | ADL/mobility residual difficulty (unmet need) (Ordered OR) | Number of children | 0.99 | - | - | Not sig |
|  | ADL/mobility residual difficulty (unmet need) (Ordered OR) | Female (ref: male) | 0.65 | - | - | <0.05 |
|  | ADL/mobility residual difficulty (unmet need) (Ordered OR) | Female*married | 1.76 | - | - | <0.05 |
|  | ADL/mobility residual difficulty (unmet need) (Ordered OR) | Married or cohabiting (Referent: no) | 0.64 | - | - | <0.05 |
|  | ADL/mobility residual difficulty (unmet need) (Ordered OR) | Black populations (referent: white) | 1.02 | - | - | Not sig |
|  | ADL/mobility residual difficulty (unmet need) (Ordered OR) | Age squared | 0.002 | - | - | 0.10 |
| Andrew 2014 | Unmet need for living (OR) | Living in a major city (ref: no) | 1.9 | 1.2 | 2.9 |  |
| Berridge 2018 | Unmet need at round 2 (factors at round 1) (coef) | Black ethnicity x any need round 1 | -0.51 | *-1.15 | 0.14 |  |
| Burchardt 2018* | Probability of receiving any care (coef) | Male (ref: female) | -0.007 | - | - | Not sig |
|  | Probability of receiving formal care (coef) | Male (ref: female) | -0.000 | - | - | Not sig |
|  | Probability of receiving informal care (coef) | Male (ref: female) | -0.027 | - | - | Not sig |
|  | UK regions, age, ethnicity: data not reported here due to volume. Reader is referred to the publication. | | | | | |
| Davey 2013 | Unmet BADL/IADL needs (with state-level home help coverage rate interaction) (coef) | Lives with child (ref: does not) | -0.57 | - | - | <0.01 |
|  | Unmet BADL/IADL needs (with state-level intensity of services rate interaction) (coef) | Lives with child (ref: does not) | -0.53 | - | - | <0.05 |
|  | Unmet BADL/IADL needs (with state-level institutionalisation rate interaction) (coef) | Lives with child (ref: does not) | -0.56 | - | - | <0.01 |
| Dunatchnik 2016 | Developing unmet ADL/IADL need at 10 years (OR) | Data not reported in publication: presence of children in house, how often respondent meets/talks to children, whether respondent thinks the family understands how he/she feels, whether respondent has friends, are not significant predictors of unmet needs. | | | | |
| Gousia 2021 | Unmet social care needs (OR) | London (Ref: South West) | 1.2 | 0.91 | 1.57 |  |
|  | Unmet social care needs (OR) | East Midlands (Ref: South West) | 1.32 | 1.02 | 1.71 |  |
|  | Unmet social care needs (OR) | West Midlands (Ref: South West) | 1.08 | 0.83 | 1.4 |  |
|  | Unmet social care needs (OR) | North East (Ref: South West) | 1.01 | 0.75 | 1.35 |  |
|  | Unmet social care needs (OR) | North West (Ref: South West) | 1.09 | 0.86 | 1.41 |  |
|  | Unmet social care needs (OR) | Yorkshire and Humber (Ref: South West) | 0.97 | 0.74 | 1.27 |  |
|  | Unmet social care needs (OR) | South East (Ref: South West) | 0.8 | 0.63 | 1.04 |  |
|  | Unmet social care needs (OR) | East of England (Ref: South West) | 0.82 | 0.64 | 1.06 |  |
| Hlebec 2015 | Unmet PADL/IADL need (OR) | Household size 3+ (ref: <3) | 2.11 | 1.1 | 4.05 |  |
|  | Unmet PADL/IADL need (OR) | Lives with spouse (ref: does not) | 1.27 | 0.66 | 2.46 |  |
|  | Unmet PADL/IADL need (OR) | Urban (ref: rural) | 0.47 | 0.24 | 0.91 |  |
| Kemper 2007 | Lacking ADL help (coef) | Number of children | 0.0013 | - | - | Not sig |
|  | Lacking ADL help (coef) | Metropolitan area (ref: no) | 0.0367 | 0.0234 |  | Not sig |
| La Plant 2004* | Probability of receiving ADL help (coef) | Non-white (ref: white) | -0.0958 | - | - | Not sig |
|  | Probability of receiving ADL help (coef) | Age (range 18-99) | -0.0001 | - | - | Not sig |
| Li 2006 | Unmet ADL needs (OR) | Urban (ref: rural) | 1.15 | 0.6 | 2.21 |  |
| Shea 2003 | Unmet ADL need (coef) | Sweden (ref: US) | -0.919 | - | - | <0.001 |
| Wilkinson-Meyers 2013 | Unmet need for assistance with ADLs and IADLs (OR) | Is a carer to others (ref: no) | 1.7 | 1.4 | 2.2 |  |
| Zhou 2018* | Number of unmet ADL need (coef) | Female (ref: male) | −0.29 | −0.53 | −0.05 |  |
|  | Number of unmet IADL need (coef) | Female (ref: male) | −0.38 | −0.73 | −0.04 |  |
|  | Number of any unmet need (coef) | Female (ref: male) | −0.32 | −0.54 | −0.09 |  |
|  | Number of unmet ADL need (coef) | Rural (ref: urban) | 0.03 | −0.21 | 0.27 |  |
|  | Number of unmet IADL need (coef) | Rural (ref: urban) | 0.1 | −0.25 | 0.46 |  |
|  | Number of any unmet need (coef) | Rural (ref: urban) | 0.07 | −0.15 | 0.3 |  |
|  | Number of unmet ADL need (coef) | Single, widowed, or divorced (ref: married) | 0.2 | −0.05 | 0.45 |  |
|  | Number of unmet IADL need (coef) | Single, widowed, or divorced (ref: married) | 0.29 | −0.07 | 0.66 |  |
|  | Number of any unmet need (coef) | Single, widowed, or divorced (ref: married) | 0.23 | −0.01 | 0.47 |  |
|  | Number of unmet ADL need (coef) | Indigenous (ref: other) | 0.1 | −0.26 | 0.46 |  |
|  | Number of unmet IADL need (coef) | Indigenous (ref: other) | 0.29 | −0.24 | 0.82 |  |
|  | Number of any unmet need (coef) | Indigenous (ref: other) | 0.2 | −0.14 | 0.55 |  |
|  | Age: data not reported here due to volume. Reader is referred to the publication. | | | | | |
| Zuverink 2020* | Unmet ADL need (HR) | Male (ref: female) | 1.08 | 0.83 | 1.41 |  |
|  | Unmet IADL need (HR) | Male (ref: female) | 1.28 | 1.03 | 1.58 |  |
|  | Unmet mobility need (HR) | Male (ref: female) | 1.25 | 0.99 | 1.58 |  |
|  | Age: data not reported here due to volume. Reader is referred to the publication. | | | | | |

^a^Referent in brackets; *Not included in plots due to type of analysis

Table S3. Socioeconomic data not included in forest plots

| **Study** | **Outcome** | **Exposure (factor)**^a^ | **Estimate** | **Lower CI** | **Upper CI** | **P** |
| --- | --- | --- | --- | --- | --- | --- |
| Agree 1999* | ADL/mobility residual difficulty (unmet need) (Ordered OR) | Fair/poor housing quality | 1.77 | - | - | <0.001 |
|  | ADL/mobility residual difficulty (unmet need) (Ordered OR) | High school, no college degree (ref: no) | 1.22 | - | - | Not sig |
|  | ADL/mobility residual difficulty (unmet need) (Ordered OR) | College degree (ref: no) | 0.98 | - | - | Not sig |
|  | ADL/mobility residual difficulty (unmet need) (Ordered OR) | Net worth <US$39,099^b^ | 0.95 | - | - |  |
|  | ADL/mobility residual difficulty (unmet need) (Ordered OR) | Net worth> US$39,099^b^ | 1.16 | - | - |  |
|  | ADL/mobility residual difficulty (unmet need) (Ordered OR) | Home value (logged dollar) | 1.02 | - | - | Not sig |
| Burchardt 2018* | Probability of receiving formal, informal and any care (coef) | Income: data not extracted due to volume. Reader referred to publication. | | | | |
| Dunatchnik 2016 | Developing unmet ADL/IADL need at 10 years (OR) | Data not reported: non-housing wealth not sig predictor of unmet needs | | | | |
| Gousia 2021 | Unmet social care needs (OR) | 2nd wealth quintile (ref: 1st) | 0.74 | 0.65 | 0.85 |  |
|  | Unmet social care needs (OR) | 3rd wealth quintiles (ref: 1st) | 0.57 | 0.49 | 0.66 |  |
|  | Unmet social care needs (OR) | 4th wealth quintiles (ref: 1st) | 0.43 | 0.37 | 0.51 |  |
|  | Unmet social care needs (OR) | 5th wealth quintiles (ref: 1st) | 0.41 | 0.34 | 0.5 |  |
| La Plant 2004* | Probability of receiving ADL help (coef) | Income below 300% of SSI (ref: no) | 0.0622 | - | - | Not sig |
| Wilkinson-Meyers 2013 | Unmet need for assistance with ADLs and IADLs (OR) | Has mortgage (ref: no) | 1.8 | 1.2 | 2.7 |  |
|  | Unmet need for assistance with ADLs and IADLs (OR) | Standard of living low/medium (ref: high) | 1.4 | 1.2 | 1.7 |  |
| Zhou 2018* | Number of unmet ADL need (coef) | Primary school (ref: no education) | -0.11 | -0.37 | 0.14 |  |
|  | Number of unmet ADL need (coef) | Secondary school (ref: no education) | -0.42 | -0.83 | -0.02 |  |
|  | Number of unmet IADL need (coef) | Primary school (ref: no education) | 0.33 | -0.06 | 0.72 |  |
|  | Number of unmet IADL need (coef) | Secondary school (ref: no education) | 0.26 | -0.31 | 0.84 |  |
|  | Number of any unmet need (coef) | Primary school (ref: no education) | 0.08 | -0.17 | 0.33 |  |
|  | Number of any unmet need (coef) | Secondary school (ref: no education) | -0.1 | -0.47 | 0.27 |  |
|  | Number of unmet ADL need (coef) | Income^c^ | 0.04 | -0.06 | 0.14 |  |
|  | Number of unmet IADL need (coef) | Income^c^ | -0.12 | -0.28 | 0.04 |  |
|  | Number of any unmet need (coef) | Income^c^ | -0.02 | -0.12 | 0.07 |  |
| Zuverink 2020* | Unmet ADL need at follow up (HR) | High school (ref: < high school) | 1.06 | 0.81 | 1.39 |  |
|  | Unmet ADL need at follow up (HR) | Some college no degree (ref: <high school) | 1.18 | 0.85 | 1.65 |  |
|  | Unmet ADL need at follow up (HR) | College degree (ref: < high school) | 1.09 | 0.74 | 1.59 |  |
|  | Unmet IADL need at follow up (HR) | High school (ref: < high school) | 0.84 | 0.72 | 1.14 |  |
|  | Unmet IADL need at follow up (HR) | Some college no degree (ref: <high school) | 0.93 | 0.67 | 1.31 |  |
|  | Unmet IADL need at follow up (HR) | College degree (ref: < high school) | 1.14 | 0.83 | 1.55 |  |
|  | Mobility unmet need at follow up (HR) | High school (ref: < high school) | 1.19 | 0.88 | 1.6 |  |
|  | Mobility unmet need at follow up (HR) | Some college no degree (ref: <high school) | 1.29 | 0.96 | 1.74 |  |
|  | Mobility unmet need at follow up (HR) | College degree (ref: < high school) | 1.58 | 1.19 | 2.08 |  |

^a^Referent in brackets; ^b^ Income is used as a set of two continuous variables that represent the logged income distribution below and above US$39, ^c^Income measured in categories but categories not reported in model; *Not included in plots due to type of analysis

| **Table S4. Health and disability data not included in forest plots** | | | | | | |
| --- | --- | --- | --- | --- | --- | --- |
| **Study** | **Outcome** | **Exposure (factor)^a^** | **Estimate** | **Lower CI** | **Upper CI** | **P** |
| Adam 1999 | Any unmet ADL need (OR) | Poor vision (ref: no) | 0.85 | 0.72 | 1.00 |  |
|  | Any unmet ADL need (OR) | Poor hearing (ref: no) | 1.18 | 1.00 | 1.39 |  |
| Agree 1999* | ADL/mobility residual difficulty (unmet need) (Ordered OR) | Chronic lung disease (ref: no) | 1.46 | - | - | <0.05 |
|  | ADL/mobility residual difficulty (unmet need) (Ordered OR) | Stroke/TIA (ref: no) | 1.09 | - | - | Not sig. |
|  | ADL/mobility residual difficulty (unmet need) (Ordered OR) | Heart disease (ref: no) | 1.33 | - | - | <0.05 |
|  | ADL/mobility residual difficulty (unmet need) (Ordered OR) | Arthritis (ref: no) | 1.33 | - | - | <0.1 |
|  | ADL/mobility residual difficulty (unmet need) (Ordered OR) | Cognitive impairment (ref: no) | 0.94 | - | - | Not sig. |
|  | ADL/mobility residual difficulty (unmet need) (Ordered OR) | Cog impairment*proxy (ref: no) | 1.77 | - | - | <0.1 |
| Burchardt 2018* | Probability of receiving any care (coef) | Vision | 0.028 | - | - | Not sig. |
|  | Probability of receiving any care (coef) | Hearing | -0.03 | - | - | Not sig. |
|  | Probability of receiving formal care (coef) | Vision | -0.016 | - | - | Not sig |
|  | Probability of receiving formal care (coef) | Hearing | -0.013 | - | - | Not sig |
|  | Probability of receiving informal care (coef) | Vision | 0.094 | - | - | 0.1 |
|  | Probability of receiving informal care (coef) | Hearing | 0.01 | - | - | Not sig |
|  | Probability of receiving informal care (coef) | Dexterity | 0.116 | - | - | 0.05 |
| Dunatchnik 2016 | ALD/IADL unmet need at ten years (OR) | ADL Needs at wave 6 (ref: no) | 14.38 | 7.48 | 27.67 |  |
|  | ALD/IADL unmet need at ten years (OR) | Data not reported in publication: poor wellbeing, cognitive functions, poor eyesight, smoking, physical activity were not significant predictors of unmet need. | | | | |
| Gousia 2021 | Unmet social care needs (OR) | Asthma (ref: no) | 1.61 | 1.36 | 1.89 |  |
|  | Unmet social care needs (OR) | Mild often pain (ref: no pain) | 2.45 | 2.06 | 2.91 |  |
|  | Unmet social care needs (OR) | Moderate often pain (ref: no pain) | 6.29 | 5.47 | 7.24 |  |
|  | Unmet social care needs (OR) | Severe often pain (ref: no pain) | 18.58 | 15.87 | 21.76 |  |
| Hermsen 2018 | Unmet need for environmental needs (OR) | Frailty (ref: no) | 1.15 | 0.48 | 2.75 |  |
|  | Unmet need for environmental needs (OR) | Anxiety symptoms (score) | 1.1 | 0.99 | 1.22 |  |
| La Plant 2004* | Probability receiving ADL help (coef) | Needs help bathing/showering (ref: no) | 1.0589 | - | - | <0.01 |
|  | Probability receiving ADL help (coef) | Needs help dressing (ref: no) | 0.9555 | - | - | <0.01 |
|  | Probability receiving ADL help (coef) | Needs help eating (ref: no) | 0.9366 | - | - | <0.01 |
|  | Probability receiving ADL help (coef) | Needs help transferring (ref: no) | 0.4935 | - | - | <0.01 |
|  | Probability receiving ADL help (coef) | Needs help toileting (ref: no) | 0.0589 | - | - | Not sig |
|  | Probability receiving ADL help (coef) | Needs help walking (ref: no) | 0.9181 | - | - | <0.01 |
|  | Probability receiving ADL help (coef) | Needs help getting outside (ref: no) | 0.7146 | - | - | <0.01 |
|  | Probability receiving ADL help (coef) | Needs help prep meals (ref: no) | 0.8415 | - | - | <0.01 |
|  | Probability receiving ADL help (coef) | Needs help shopping for groceries (ref: no) | 0.9635 | - | - | <0.01 |
|  | Probability receiving ADL help (coef) | Needs help managing money (ref: no) | 0.6534 | - | - | <0.01 |
|  | Probability receiving ADL help (coef) | Needs help using the phone (ref: no) | 0.6671 | - | - | <0.01 |
|  | Probability receiving ADL help (coef) | Needs help doing heavy housework (ref: no) | 0.7847 | - | - | <0.01 |
|  | Probability receiving ADL help (coef) | Needs help light housework (ref: no) | 0.6374 | - | - | <0.01 |
|  | Probability receiving ADL help (coef) | Needs help getting to places outside home (ref: no) | 1.21 | - | - | <0.01 |
|  | Probability receiving ADL help (coef) | Needs help managing medications (ref: no) | 0.8828 | - | - | <0.01 |
|  | Probability receiving ADL help (coef) | Needs help with multiple activities (ref: no) | -0.3979 | - | - | <0.01 |
|  | Probability receiving ADL help (coef) | Cognitive symptoms (ref: no) | 0.0127 | - | - | Not sig |
| Liem 2004 | Unmet need for ADL (OR) | Constipation (ref: no) | 1.97 | 1.19 | 3.26 |  |
|  | Unmet need for ADL (OR) | Years post-injury (per 10y) | 1.42 | 1.00 | 2.01 |  |
| Potter 2019 | Self-care and/or mobility unmet needs: women (OR) | Diabetes(ref: no) | 0.99 | 0.80 | 1.24 |  |
|  | Self-care and/or mobility unmet needs: women (OR) | Heart attack (ref: no) | 1.1 | 0.8 | 1.5 |  |
|  | Self-care and/or mobility unmet needs: women (OR) | Heart disease (ref: no) | 1.12 | 0.9 | 1.4 |  |
|  | Self-care and/or mobility unmet needs: women (OR) | Lung disease (ref: no) | 1.07 | 0.85 | 1.35 |  |
|  | Self-care and/mobility unmet needs: men (OR) | Diabetes (ref: no) | 1.07 | 0.78 | 1.47 |  |
|  | Self-care and/mobility unmet needs: men (OR) | Heart attack (ref: no) | 1.24 | 0.83 | 1.87 |  |
|  | Self-care and/mobility unmet needs: men (OR) | Heart disease (ref: no) | 1.1 | 0.8 | 1.52 |  |
|  | Self-care and/mobility unmet needs: men (OR) | Lung disease (ref: no) | 0.92 | 0.64 | 1.31 |  |
| Siegal 1991 | Unmet ADL/IADL need (beta coef) | Metastatic (ref: no) | 0.01 | - | - | Not sig. |
|  | Unmet ADL/IADL need (beta coef) | Radiation only (ref: chemotherapy only) | -0.386 | - | - | Not sig. |
|  | Unmet ADL/IADL need (beta coef) | Chemotherapy and radiation (ref: chemotherapy only) | 0.553 | - | - | Not sig. |
|  | Unmet ADL/IADL need (beta coef) | Other health problems (ref: no) | 0.292 | - | - | Not sig. |
|  | Unmet ADL/IADL need (beta coef) | Reduced activity days in <2 weeks 1-13 days (ref: 1 day) | 1.045 | - | - | Not sig. |
|  | Unmet ADL/IADL need (beta coef) | Reduced activity days in <2 weeks 14 days (ref: 1 day) | 1.466 | - | - | Not sig. |
|  | Unmet ADL/IADL need (beta coef) | Number of symptoms 1-2 (ref: 0) | 0.606 | - | - | Not sig. |
|  | Unmet ADL/IADL need (beta coef) | Number of symptoms 3+ (ref: 0) | 1.05 | - | - | Not sig. |
|  | Unmet ADL/IADL need (beta coef) | Patient applied for/receives PA, SSD, and/or Medicaid (ref: no) | 0.686 | - | - | Not sig. |
| Wilkinson-Meyers 2013 | Unmet need for ADL/IADL assistance (OR) | Memory difficulty (ref: no) | 1.20 | 1.00 | 1.50 |  |
| Zhou 2018* | Number of unmet ADL needs (beta coef) | MMSE Score | -0.01 | -0.05 | 0.03 |  |
|  | Number of unmet IADL needs (beta coef) | MMSE Score | -0.03 | -0.09 | 0.03 |  |
|  | Number of all unmet needs (beta coef) | MMSE Score | -0.02 | -0.06 | 0.02 |  |
| Zuverink 2020* | Unmet ADL need at follow up (HR) | Possible dementia (ref: no dementia) | 0.91 | 0.64 | 1.30 |  |
|  | Unmet ADL need at follow up (HR) | Probably dementia (ref: no dementia) | 1.04 | 0.77 | 1.41 |  |
|  | Unmet IADL needs at follow up (HR) | Possible dementia (ref: no dementia) | 0.92 | 0.66 | 1.30 |  |
|  | Unmet IADL needs at follow up (HR) | Probably dementia (ref: no dementia) | 0.94 | 0.70 | 1.27 |  |
|  | Unmet mobility needs at follow up (HR) | Possible dementia (ref: no dementia) | 1.05 | 0.76 | 1.44 |  |
|  | Unmet mobility needs at follow up (HR) | Probably dementia (ref: no dementia) | 0.91 | 0.70 | 1.18 |  |
| ^a^Referent in brackets; *Not included in plots due to type of analysis | | | | | | |

**Table S5. Health Service Use data not included in forest plots**

| **Study** | **Outcome** | **Exposure (factor)**^a^ | **Estimate** | **Lower CI** | **Upper CI** | **P** |
| --- | --- | --- | --- | --- | --- | --- |
| Adam 1999* | Any unmet ADL need (OR) | GP visit (ref: no) | 1.19 | 0.96 | 1.47 |  |
|  | Any unmet ADL need (OR) | Home visit by health professional (ref: no) | 0.86 | 0.72 | 1.03 |  |
|  | Any unmet ADL need (OR) | Dental visit (ref: no) | 1.27 | 1.05 | 1.53 |  |
| Berridge 2018 | Adverse consequences of unmet ADL need (OR) | Hospital stay in past 12 months (ref: no) | 1.14 | 0.84 | 1.55 |  |
|  | Adverse consequences of unmet IADL need (OR) | Hospital stay in past 12 months (ref: no) | 0.93 | 0.71 | 1.23 |  |
|  | Adverse consequences of any unmet need (OR) | Hospital stay in past 12 months (ref: no) | 1.43 | 1.13 | 1.81 |  |
|  | Log odds of unmet need at round 2 (coef) | Hospital stay in past 12 months (ref: no) | 0.12 | -0.1 | 0.33 |  |
| Zuverink 2020* | Unmet ADL need at follow up (HR) | Past year hospitalisation (ref: no) | 1.03 | 0.83 | 1.29 |  |
|  | Unmet IADL need at follow up (HR) | Past year hospitalisation (ref: no) | 1.22 | 0.92 | 1.63 |  |
|  | Mobility unmet need at follow up (HR) | Past year hospitalisation (ref: no) | 0.97 | 0.78 | 1.21 |  |

^a^Referent in brackets; *Not included in plots due to type of analysis

**Table S6. Care configurations data**

| **Study** | **Outcome** | **Exposure (factor)^a^** | **Estimate** | **Lower CI** | **Upper CI** | **p** |
| --- | --- | --- | --- | --- | --- | --- |
| Adam 1999 | Unmet ADL need (OR) | Has spouse (ref: no) | 0.52 | 0.43 | 0.63 | <0.001 |
|  |  | Has child nearby (ref: no) | 0.76 | 0.64 | 0.91 | <0.01 |
| Agree 1999* | Unmet ADL/mobility need (Ordered OR) | Receives personal care only (ref: equipment only) | 1.41 | - | - | <0.1 |
|  |  | Receives personal care and equipment (ref: equipment only) | 3.10 | - | - | <0.001 |
| Beach 2016 | Any unmet need (OR) | Has supplement paid help (ref: no) | 1.45 | 0.94 | 2.23 |  |
|  | Any ADL or mobility unmet need (OR) | Has supplement paid help (ref: no) | 1.12 | 0.71 | 1.77 |  |
|  | Any IADL unmet need (OR) | Has supplement paid help (ref: no) | 2.84 | 1.77 | 4.57 |  |
|  | Any unmet need (OR) | Spouse (ref: daughter, daughter in law,, step daughter) | 0.64 | 0.37 | 1.1 |  |
|  | Any unmet need (OR) | Son, son in law, step son (ref: daughter, daughter in law, step daughter) | 1.21 | 0.72 | 2.04 |  |
|  | Any unmet need (OR) | Other (ref: daughter, daughter in law, step daughter) | 1.07 | 0.7 | 1.63 |  |
|  | Any ADL or mobility unmet need (OR) | Spouse (ref: daughter, daughter in law, step daughter) | 0.66 | 0.38 | 1.14 |  |
|  | Any ADL or mobility unmet need (OR) | Son, son in law, step son (ref: daughter, daughter in law, step daughter) | 1.34 | 0.76 | 2.36 |  |
|  | Any ADL or mobility unmet need (OR) | Other (ref: daughter, daughter in law,, step daughter) | 0.93 | 0.59 | 1.46 |  |
|  | Any IADL unmet need (OR) | Spouse (ref: daughter, daughter in law, step daughter) | 1.01 | 0.46 | 2.21 |  |
|  | Any IADL unmet need (OR) | Son, son in law, step son (ref: daughter, daughter in law, step daughter) | 2.88 | 1.3 | 6.34 |  |
|  | Any IADL unmet need (OR) | Other (ref: daughter, daughter in law, step daughter) | 1.53 | 0.95 | 2.47 |  |
| Brimblecombe 2017 | Carer’s perceived unmet need for services (OR) | 10+ unpaid care hours/week (ref: <10 hours) | 1.446 | 0.564 | 3.71 | 0.443 |
|  | Care recipient’s perceived unmet need for services (OR) | 10+ unpaid care hours/week (ref: <10 hours) | 4.035 | 1.5 | 10.855 | 0.006 |
|  | Carer’s perceived unmet need for services (OR) | Care-recipient is parent/in-law (ref: other than parent in-law) | 3.289 | 1.335 | 8.105 |  |
|  | Care recipient’s perceived unmet need for services (OR) | Care-recipient is parent/in-law (ref: other than parent in-law) | 1.294 | 0.542 | 3.094 |  |
| Casado 2011 | Unmet need for community based programmes (OR) | Receives substitute help | 0.45 | 0.31 | 0.65 | < 0.001 |
|  | Unmet need for home based programmes (OR) | Receives substitute help | 0.34 | 0.2 | 0.57 | < 0.001 |
|  | Unmet need for housework services (OR) | Receives substitute help | 0.37 | 0.24 | 0.56 | < 0.001 |
|  | Unmet need for Home delivered meals (OR) | Receives substitute help | 0.56 | 0.36 | 0.87 | < 0.05 |
|  | Unmet need for transport (OR) | Receives substitute help | 0.56 | 0.35 | 0.87 | < 0.05 |
|  | Unmet need for community based programmes (OR) | Receives family support | 0.47 | 0.33 | 0.68 | <0.001 |
|  | Unmet need for home based programmes (OR) | Receives family support | 0.6 | 0.35 | 1.03 |  |
|  | Unmet need for housework services (OR) | Receives family support | 0.79 | 0.51 | 1.22 |  |
|  | Unmet need for Home delivered meals (OR) | Receives family support | 0.62 | 0.39 | 0.98 | < 0.05 |
|  | Unmet need for transport (OR) | Receives family support | 1.02 | 0.62 | 1.68 |  |
|  | Unmet need for community based programmes (OR) | Has formal help (ref: no) | 1.04 | 0.69 | 1.57 |  |
|  | Unmet need for home based programmes (OR) | Has formal help (ref: no) | 0.93 | 0.43 | 1.99 |  |
|  | Unmet need for housework services (OR) | Has formal help (ref: no) | 1.03 | 0.62 | 1.72 |  |
|  | Unmet need for Home delivered meals (OR) | Has formal help (ref: no) | 0.64 | 0.37 | 1.12 |  |
|  | Unmet need for transport (OR) | Has formal help (ref: no) | 0.94 | 0.55 | 1.61 |  |
|  | Unmet need for community based programmes (OR) | Spouse (ref: child/grandchild) | 1.11 | 0.52 | 2.35 |  |
|  | Unmet need for home based programmes (OR) | Spouse (ref: child/grandchild) | 1.59 | 0.54 | 4.73 |  |
|  | Unmet need for housework services (OR) | Spouse (ref: child/grandchild) | 2.12 | 0.85 | 5.27 |  |
|  | Unmet need for Home delivered meals (OR) | Spouse (ref: child/grandchild) | 1.57 | 0.60 | 4.12 |  |
|  | Unmet need for transport (OR) | Spouse (ref: child/grandchild) | 1.65 | 0.62 | 4.43 |  |
| Casado 2012 | Unmet need for adult day care (OR) | Spouse (ref: non-spouse) | 0.05 | 0.01 | 0.3 |  |
|  | Unmet need for personal care (OR) | Spouse (ref: non-spouse) | 0.51 | 0.14 | 1.81 |  |
|  | Unmet need for home care (OR) | Spouse (ref: non-spouse) | 0.32 | 0.09 | 1.14 |  |
|  | Unmet need for housekeeping (OR) | Spouse (ref: non-spouse) | 0.54 | 0.18 | 1.63 |  |
|  | Unmet need for transport (OR) | Spouse (ref: non-spouse) | 0.37 | 0.11 | 1.23 |  |
| Kennedy 2001 | Unmet or undermet ADL/IADL need (OR) | Assistant payment status: paid care *only* (ref: unpaid only) | 0.80 | 0.50 | 1.00 |  |
|  | Unmet or undermet ADL/IADL need (OR) | Assistant payment status: paid and unpaid (ref: unpaid only) | 0.60 | 0.30 | 0.90 |  |
| Li 2006 | Unmet ADL need (OR) | Receives informal care *only* | 1.19 | 0.54 | 2.61 |  |
|  | Unmet ADL need (OR) | Receives formal and informal care (ref: does not receive both) | 1.33 | 0.3 | 5.97 |  |
|  | Unmet ADL need (OR) | Total hours of formal and informal ADL assistance | 0.71 | 0.54 | 0.95 |  |
|  | Unmet ADL need (OR) | % formal care hours | 0.82 | 0.38 | 1.79 |  |
| Potter 2019 | Unmet needs self-care/mobility, women (OR) | One non-spouse caregiver (ref: spouse only) | 1.29 | 0.93 | 1.79 |  |
|  | Unmet needs self-care/mobility, women (OR) | Spouse and other caregivers (ref: spouse only) | 1.78 | 1.22 | 2.61 |  |
|  | Unmet needs self-care/mobility, women (OR) | Multiple caregivers no spouse (ref: spouse only) | 1.87 | 1.35 | 2.6 |  |
|  | Unmet needs self-care/mobility, women (OR) | % care that is informal | 0.81 | 0.57 | 1.15 |  |
|  | Unmet needs self-care/mobility, women (OR) | Monthly care hours 10-50 (ref: 0-10) | 0.79 | 0.48 | 1.3 |  |
|  | Unmet needs self-care/mobility, women (OR) | Monthly care hours 50-100 (ref: 0-10) | 0.80 | 0.48 | 1.33 |  |
|  | Unmet needs self-care/mobility, women (OR) | Monthly care hours 100-250 (ref: 0-10) | 1.02 | 0.63 | 1.67 |  |
|  | Unmet needs self-care/mobility, women (OR) | Monthly care hours 250+ (ref: 0-10) | 0.97 | 0.59 | 1.6 |  |
|  | Unmet needs self-care/mobility, women (OR) | Multiple carers (ref: one carer) | 1.55 | 1.27 | 1.88 |  |
|  | Unmet needs for self-care/mobility, men (OR) | One non-spouse caregiver (ref: spouse only) | 1.96 | 1.28 | 3.00 |  |
|  | Unmet needs for self-care/mobility, men (OR) | Spouse and other caregivers (ref: spouse only) | 2.13 | 1.44 | 3.17 |  |
|  | Unmet needs for self-care/mobility, men (OR) | Multiple caregivers no spouse (ref: spouse only) | 1.56 | 1.00 | 2.44 |  |
|  | Unmet needs for self-care/mobility, men (OR) | % care that is informal | 1.23 | 0.62 | 2.41 |  |
|  | Unmet needs for self-care/mobility, men (OR) | Monthly care hours 10-50 (ref: 0-10) | 0.83 | 0.4 | 1.76 |  |
|  | Unmet needs for self-care/mobility, men (OR) | Monthly care hours 50-100 (ref: 0-10) | 0.82 | 0.39 | 1.73 |  |
|  | Unmet needs for self-care/mobility, men (OR) | Monthly care hours 100-250 (ref: 0-10) | 0.76 | 0.37 | 1.56 |  |
|  | Unmet needs for self-care/mobility, men (OR) | Monthly care hours 250+ (ref: 0-10) | 1.14 | 0.55 | 2.35 |  |
|  | Unmet needs for self-care/mobility, men (OR) | Multiple carers (ref: one carer) | 1.46 | 1.06 | 2.02 |  |
| Siegal 1991 | Unmet ADL/IADL need (beta coef) | Other relative (ref: spouse) | 0.962 | - | - | <0.05 |
|  | Unmet ADL/IADL need (beta coef) | Non-relative (ref: spouse) | 1.649 | - | - | <0.01 |
| Tennestedt 1995 | Unmet IADL at baseline (OR) | Caregiver relationship offspring (ref: spouse) | 3.11 | 1.11 | 8.73 |  |
|  | Unmet IADL at baseline (OR) | Caregiver relationship non-relative (ref: spouse) | 6.35 | 1.61 | 25.00 |  |
|  | Unmet IADL at follow up (OR) | Caregiver relationship: spouse (ref: non relative) | 0.04 | 0.005 | 0.34 |  |
|  | Unmet IADL at follow up (OR) | Caregiver relationship offspring (ref: non-relative): | 0.18 | 0.075 | 0.45 |  |
|  | Unmet IADL at baseline (OR) | Number of caregivers | 0.75 | 0.58 | 0.96 |  |
|  | Unmet IADL at follow up (OR) | Number of caregivers | 0.71 | 0.5 | 1.01 |  |
|  | Unmet PADL needs at follow up (OR) | Change from informal to formal care | 4.90 | 1.47 | 16.40 |  |
| Zhou 2018* | Number of all unmet needs, score (beta coef) | Care time provided | 0.06 | -0.06 | 0.18 | 0.32 |
|  | Number of unmet ADL needs, score (beta coef) | Care time provided | 0.01 | -0.16 | 0.17 | 0.94 |
|  | Number of unmet IADL needs, score (beta coef) | Care time provided | 0.03 | -0.07 | 0.14 | 0.54 |
|  | Number of all unmet needs, score (beta coef) | Number of caregivers | -0.31 | -0.59 | -0.30 |  |
|  | Number of unmet ADL needs, score (beta coef) | Number of caregivers | -0.39 | -0.70 | -0.07 |  |
|  | Number of unmet IADL needs, score (beta coef) | Number of caregivers | -0.24 | -0.67 | 0.18 |  |

**^a^**Referent in brackets; *Not included in plots due to type of analysis

Table S7. Unpaid carer characteristics

| **Study** | **Outcome** | **Exposure (factor)^a^** | **Estimate** | **Lower CI** | **Upper CI** | **p** |
| --- | --- | --- | --- | --- | --- | --- |
| Beach 2016 | Any unmet need (OR) | Caregiver lives with recipient (ref: no) | 1.09 | 0.78 | 1.52 |  |
|  | Any ADL or mobility unmet need (OR) | Caregiver lives with recipient (ref: no) | 1.10 | 0.82 | 1.48 |  |
|  | Any IADL unmet need (OR) | Caregiver lives with recipient (ref: no) | 0.65 | 0.38 | 1.10 |  |
| Casado 2012 | Unmet need for adult day care (OR) | Caregiver burden^b^ | 1.06 | 0.96 | 1.17 |  |
|  | Unmet need for personal care (OR) | Caregiver burden^b^ | 1.06 | 0.98 | 1.13 |  |
|  | Unmet need for home care (OR) | Caregiver burden^b^ | 1.06 | 0.98 | 1.14 |  |
|  | Unmet need for housekeeping (OR) | Caregiver burden^b^ | 1.03 | 0.96 | 1.1 |  |
|  | Unmet need for transport (OR) | Caregiver burden^b^ | 0.99 | 0.92 | 1.06 |  |
|  | Unmet need for adult day care (OR) | Caregiver duration (years) | 1.15 | 1.02 | 1.29 |  |
|  | Unmet need for personal care (OR) | Caregiver duration (years) | 1.00 | 0.93 | 1.08 |  |
|  | Unmet need for home care (OR) | Caregiver duration (years) | 1.01 | 0.94 | 1.09 |  |
|  | Unmet need for housekeeping (OR) | Caregiver duration (years) | 1.01 | 0.94 | 1.09 |  |
|  | Unmet need for transport (OR) | Caregiver duration (years) | 1.05 | 0.97 | 1.14 |  |
| Seigal 1991 | Unmet ADL/IADL need (beta coef) | Caregiver burden^b^ | 0.034 | - | - | Not sig |
|  | Unmet ADL/IADL need (coef) | Number of domains of patient need for which caregiver is providing assistance | -0 .238 | - | - | <0.001 |
|  | Unmet ADL/IADL need (coef) | Interaction: burden*domains | 0.130 | - | - | <0.01 |
|  | Unmet ADL/IADL need (coef) | Caregiver age | 0.007 | - | - | Not sig |
|  | Unmet ADL/IADL need (coef) | Caregiver sex^c^ | -0.550 | - | - | Not sig |
|  | Unmet ADL/IADL need (coef) | Caregiver some college education (ref: high school) | 0.230 | - | - | Not sig |
|  | Unmet ADL/IADL need (coef) | Caregiver college graduate (ref: high school | -0.062 | - | - | Not sig |
|  | Unmet ADL/IADL need (coef) | Caregiver has limiting condition (ref: no) | 0.384 | - | - | Not sig |
|  | Unmet ADL/IADL need (coef) | Caregiver number of conflicting roles | 0.084 | - | - | Not sig |

**^a^**Referent in brackets; ^b^Higher scores=greater burden; ^c^Referent not reported in the publication

Table S8. Area-level measures

| **Study** | **Exposure (factor)** | **Coef of unmet need** | **95% CI/p value** |
| --- | --- | --- | --- |
| Davey 2013 | Home help coverage rate, state-level | 0.06 | Not significant^a^ |
|  | Home help intensity level, state-level | -0.15 | Not significant^a^ |
|  | % of population in care homes, state-level | -0.26 | Not significant^a^ |

^a^ CI and p level not reported.

# Figures

**Notes and abbreviations for Figures 2a-5m**

Measure: describes unmet need measure (e.g. absolute, relative)

- *Absolute/relative: measure was one that combined absolute and relative unmet need*

Outcome: describes type of unmet need (e.g. unmet ADL need)

- ADL/IADL/mobility: measure combines type of unmet need

ADL: Activities of Daily Living

IADL: Instrumental Activities of Daily Living

BADL: Basic Activities of Daily Living

PADL: Physical Activities of Daily Living

HDM: Home delivered meals

HWS: Housework Services

HBP: Home Based Programmes

CBP: Community based Programmes

TRS: Transport Service

Figure 2a. Age and odds of unmet need


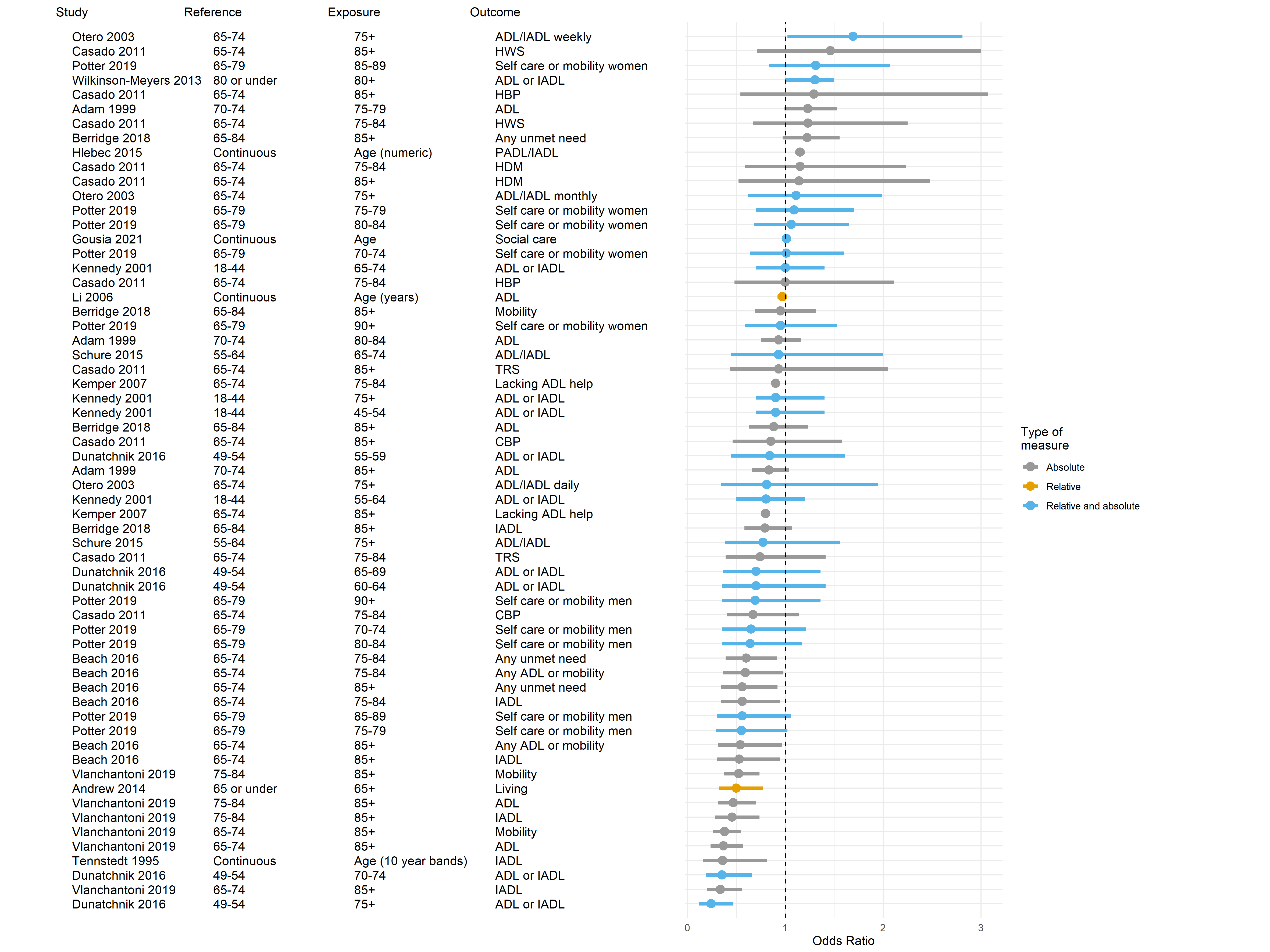


Figure 2b. Sex and odds of unmet need


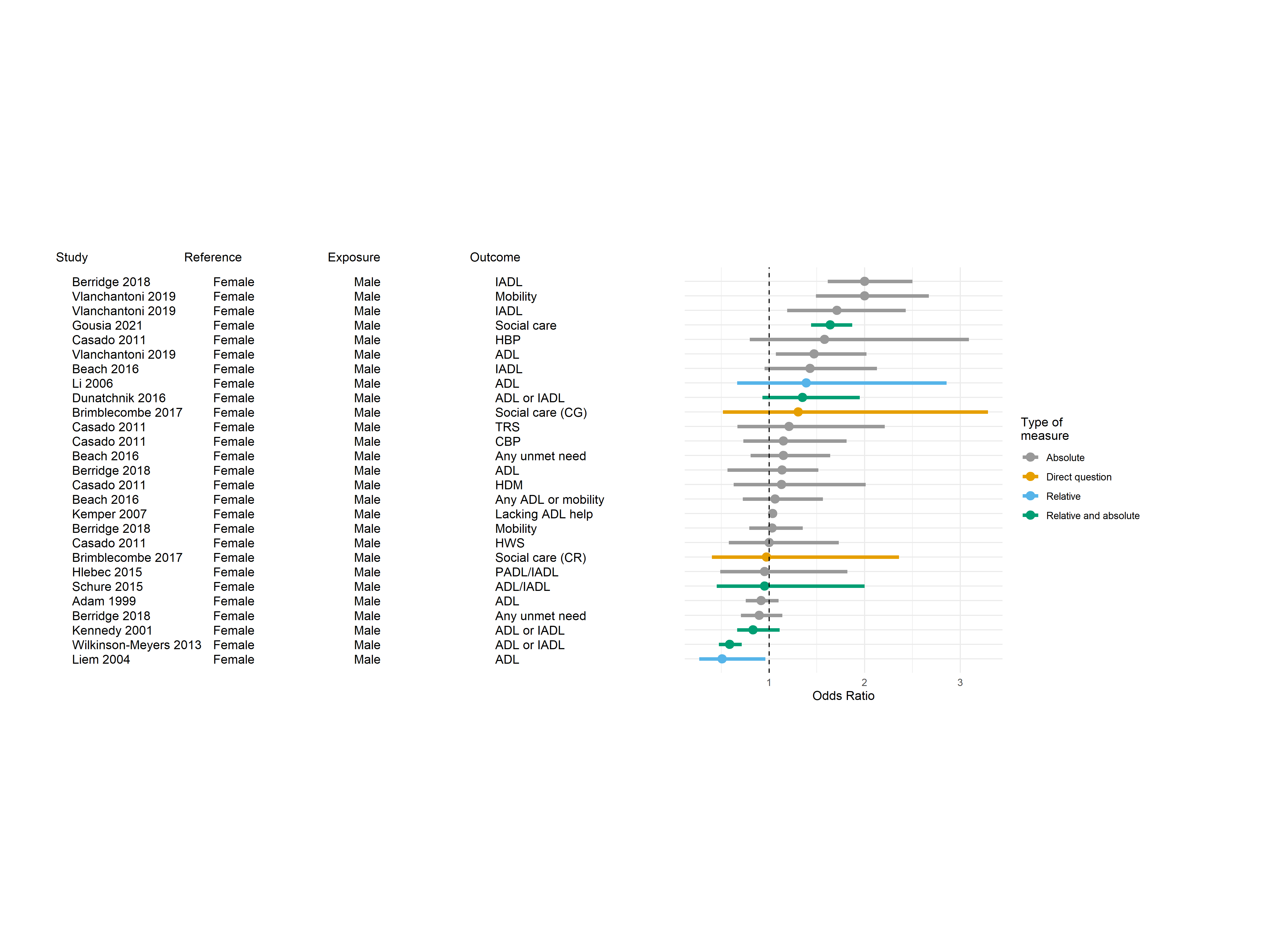


Figure 2c. Living arrangements and odds of unmet need


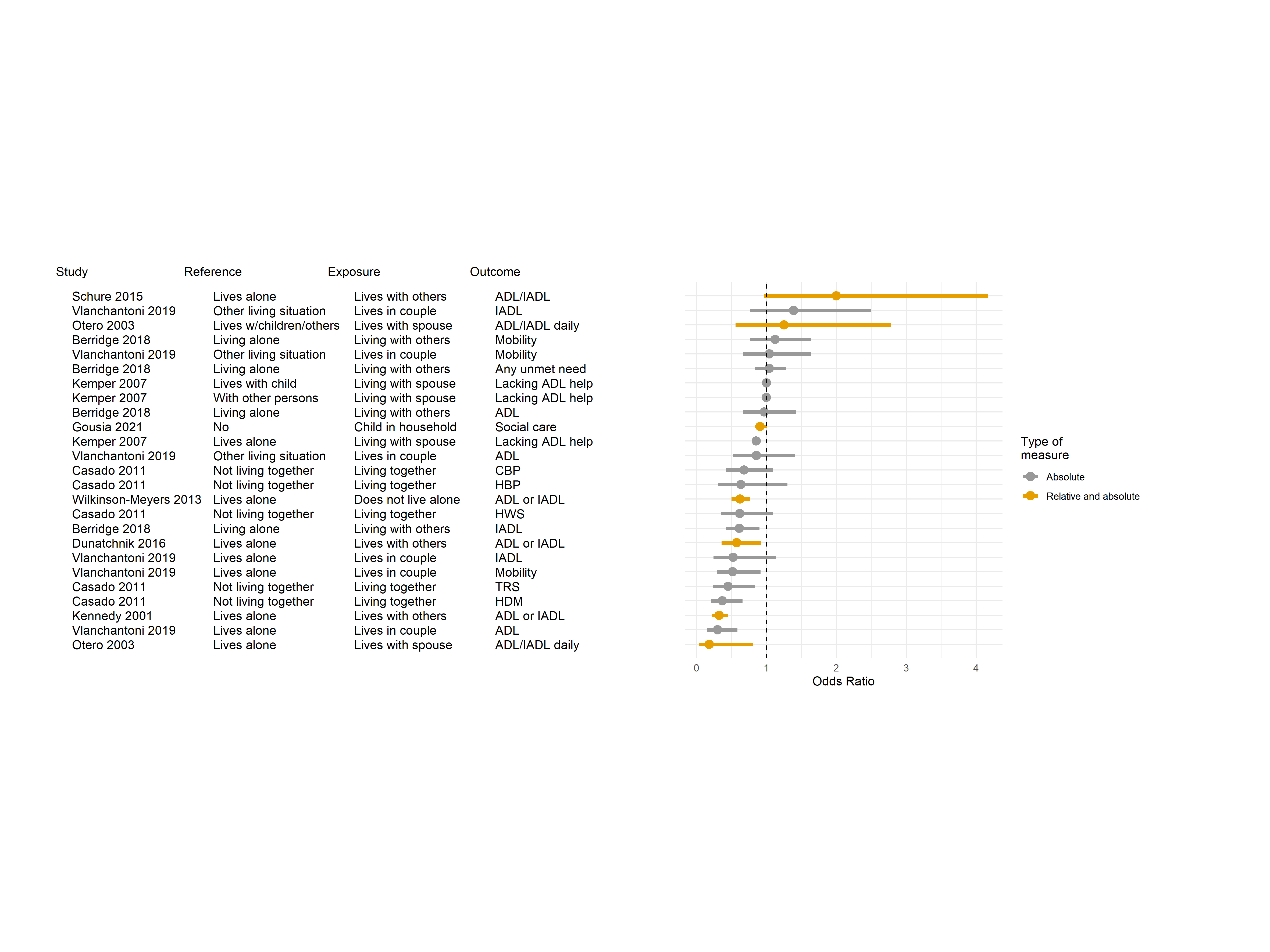


Figure 2d. Marital status and odds of unmet need


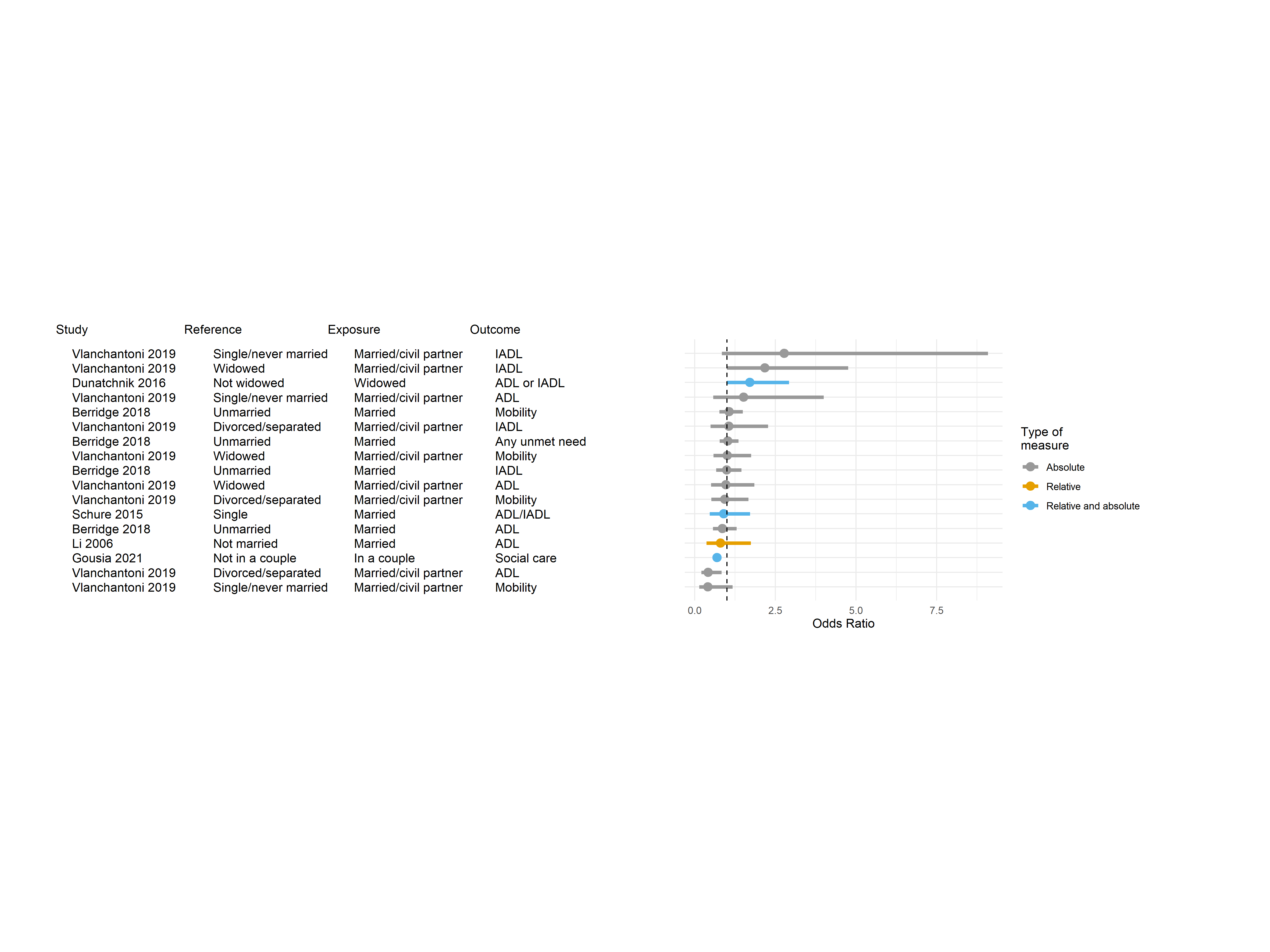


Figure 2e. Ethnicity and odds of unmet need


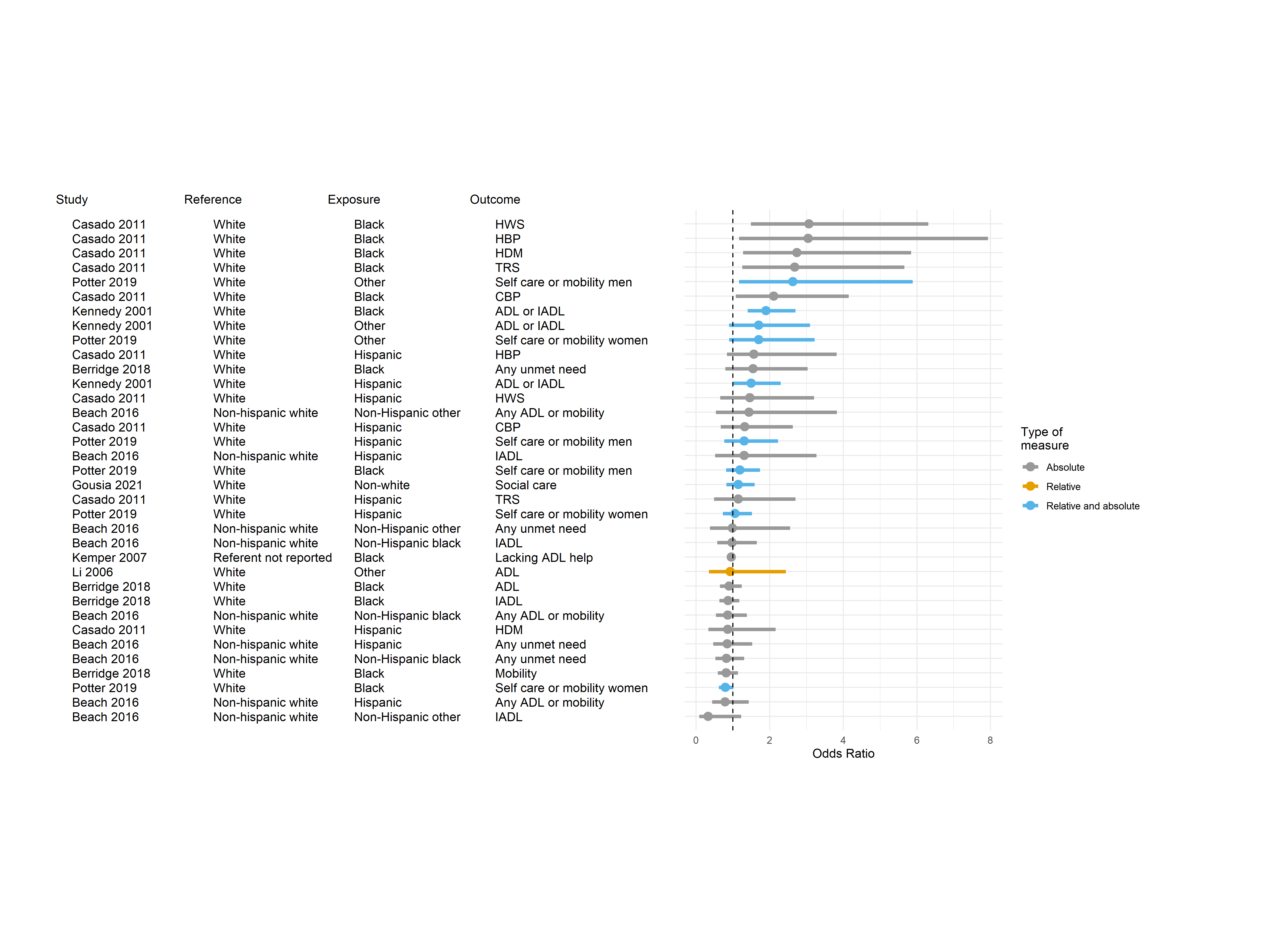


Figure 3a. Education and odds of unmet need


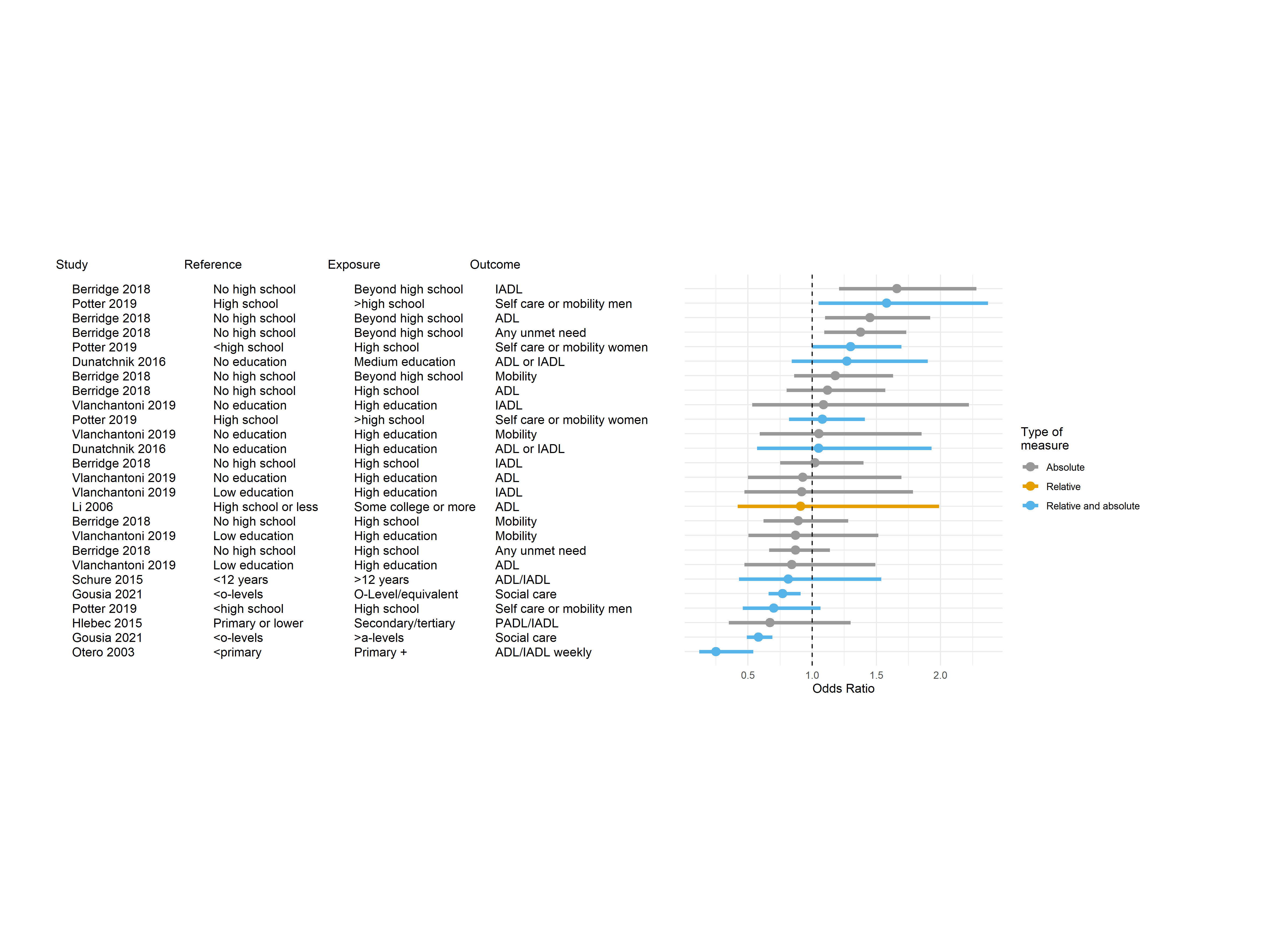


Figure 3b. Occupation and odds of unmet need


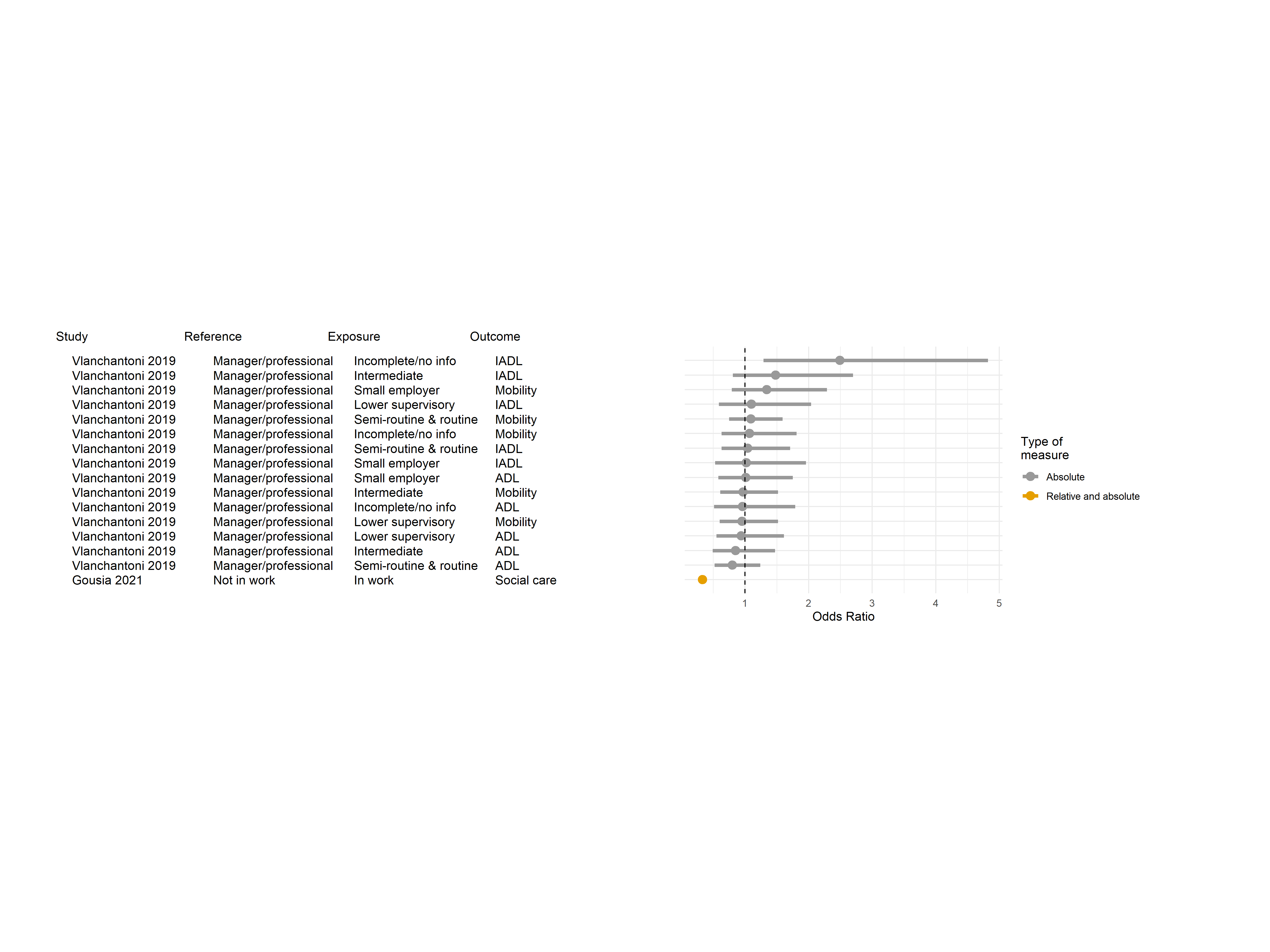


Figure 3c. Income and odds of unmet need


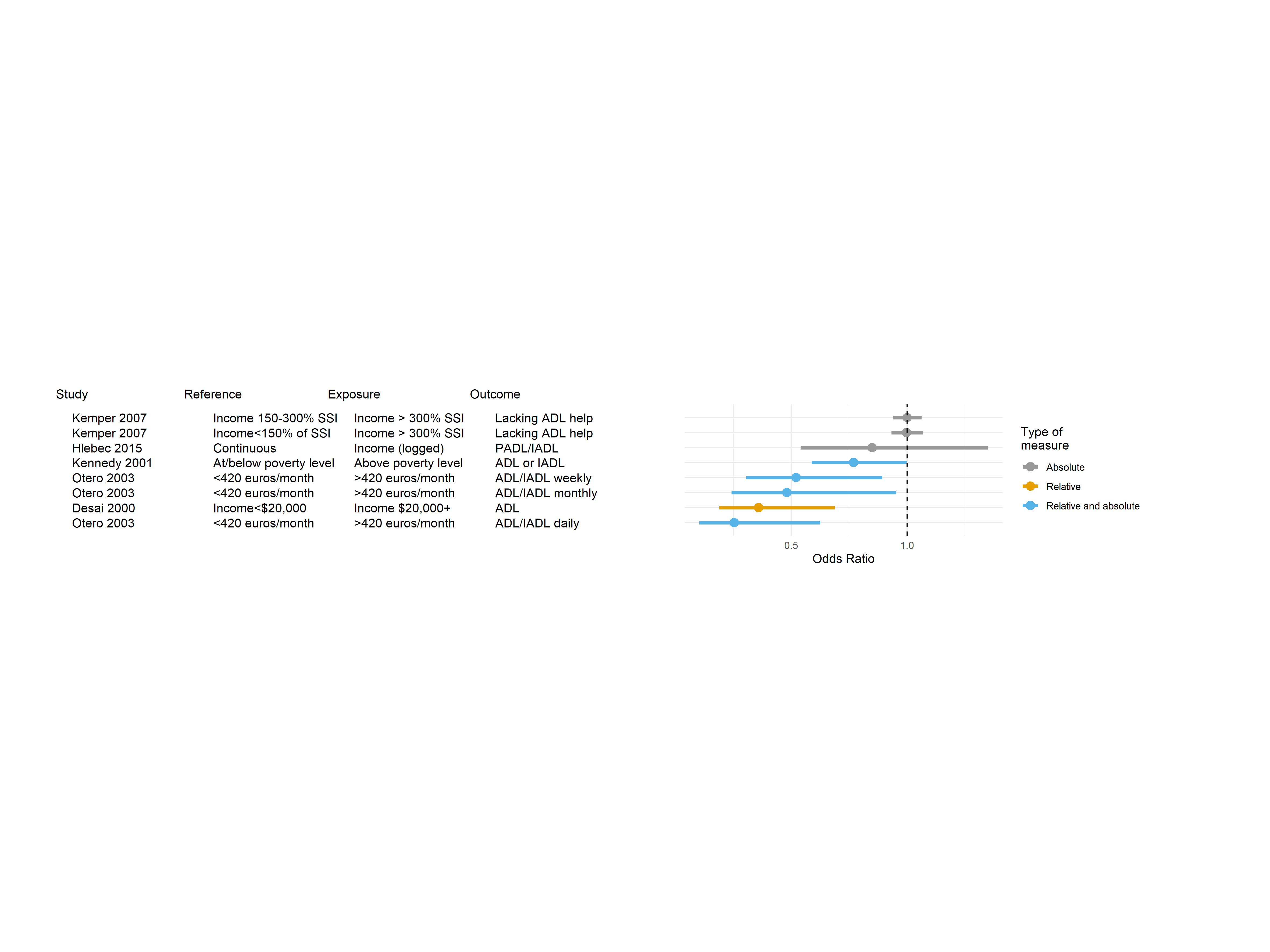


Figure 3d. Housing tenure and odds of unmet need


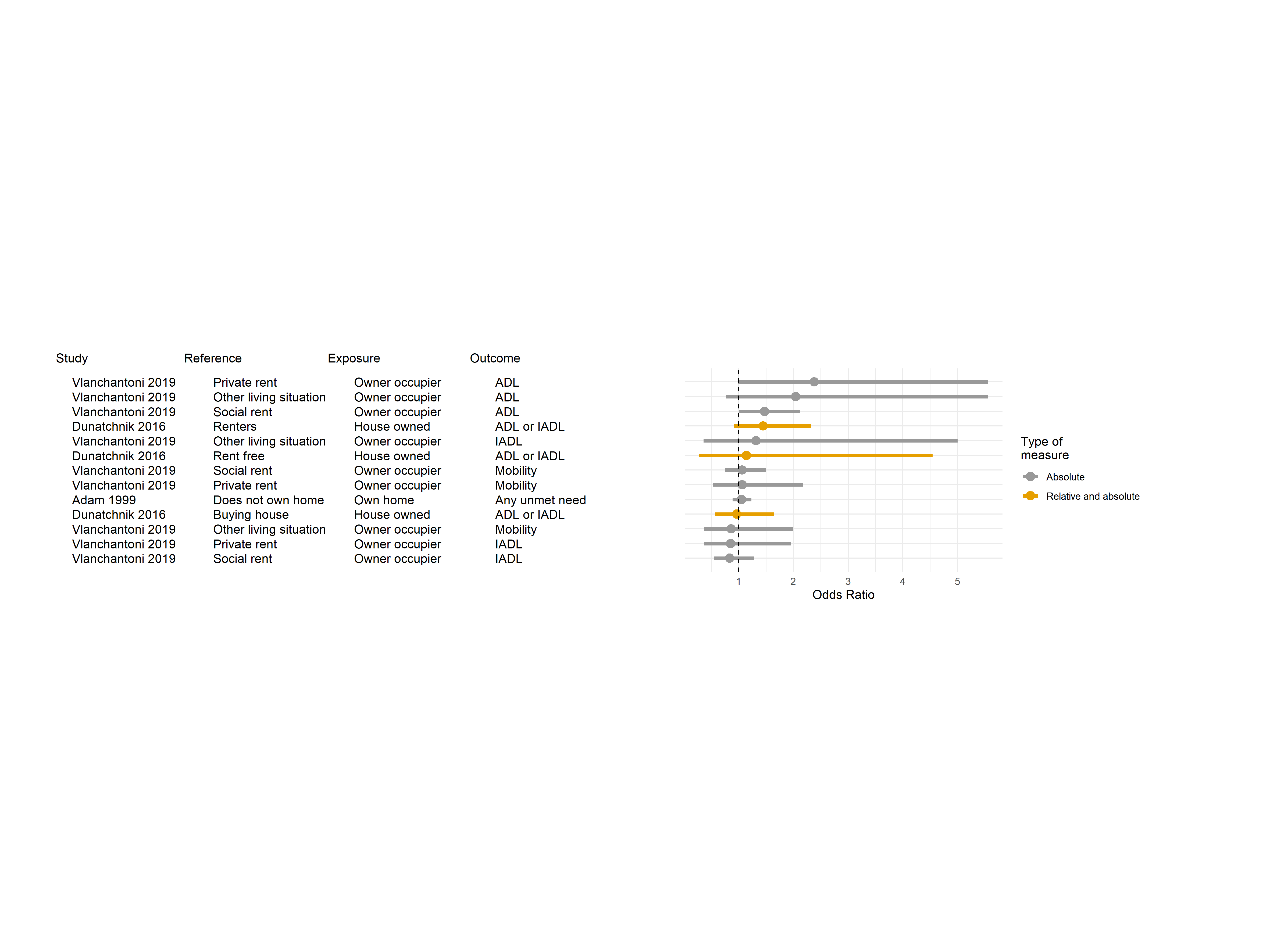


Figure 3e. Medicaid recipient and odds of unmet need


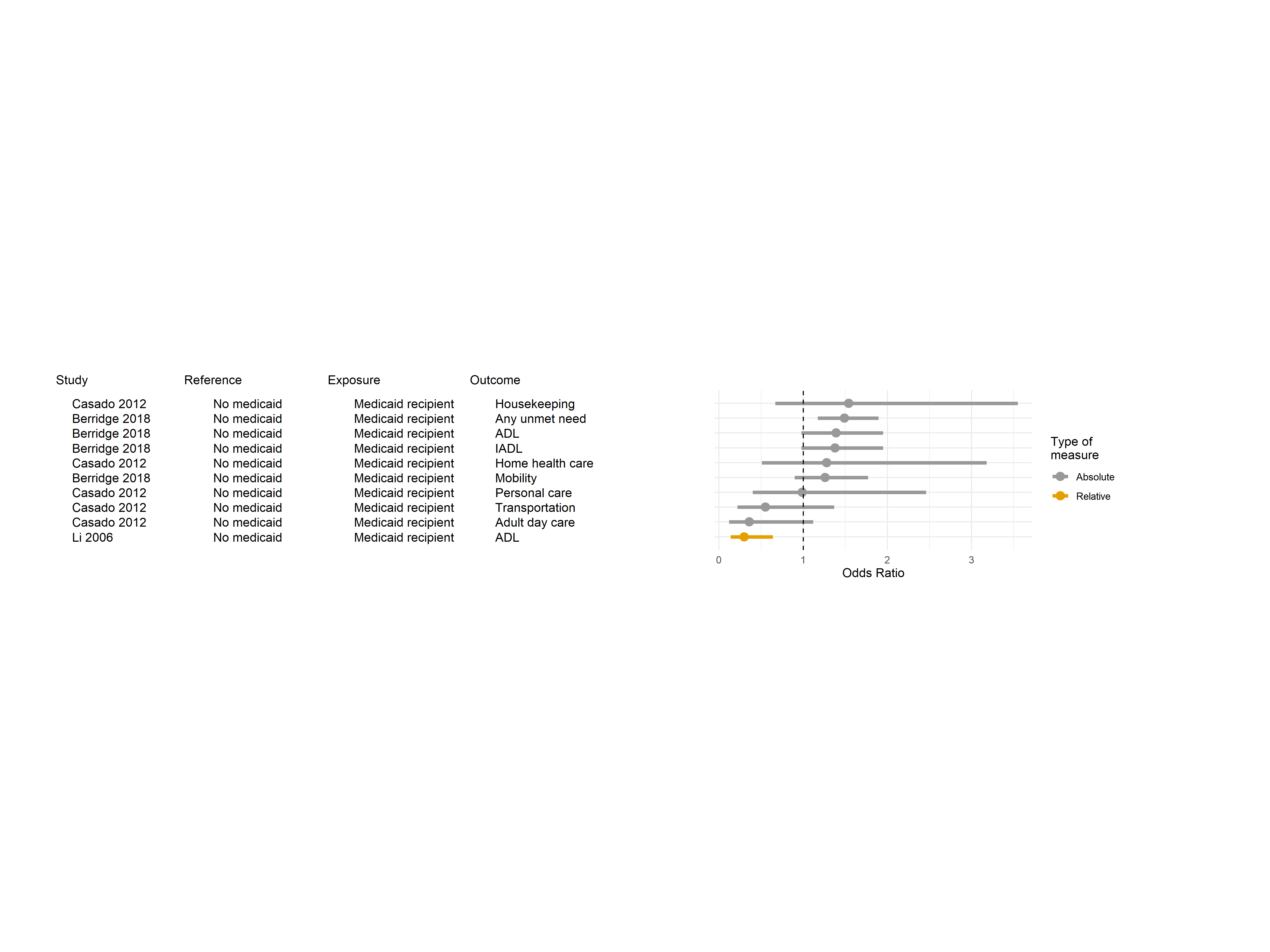


Figure 4a. Self-rated health and odds of unmet need


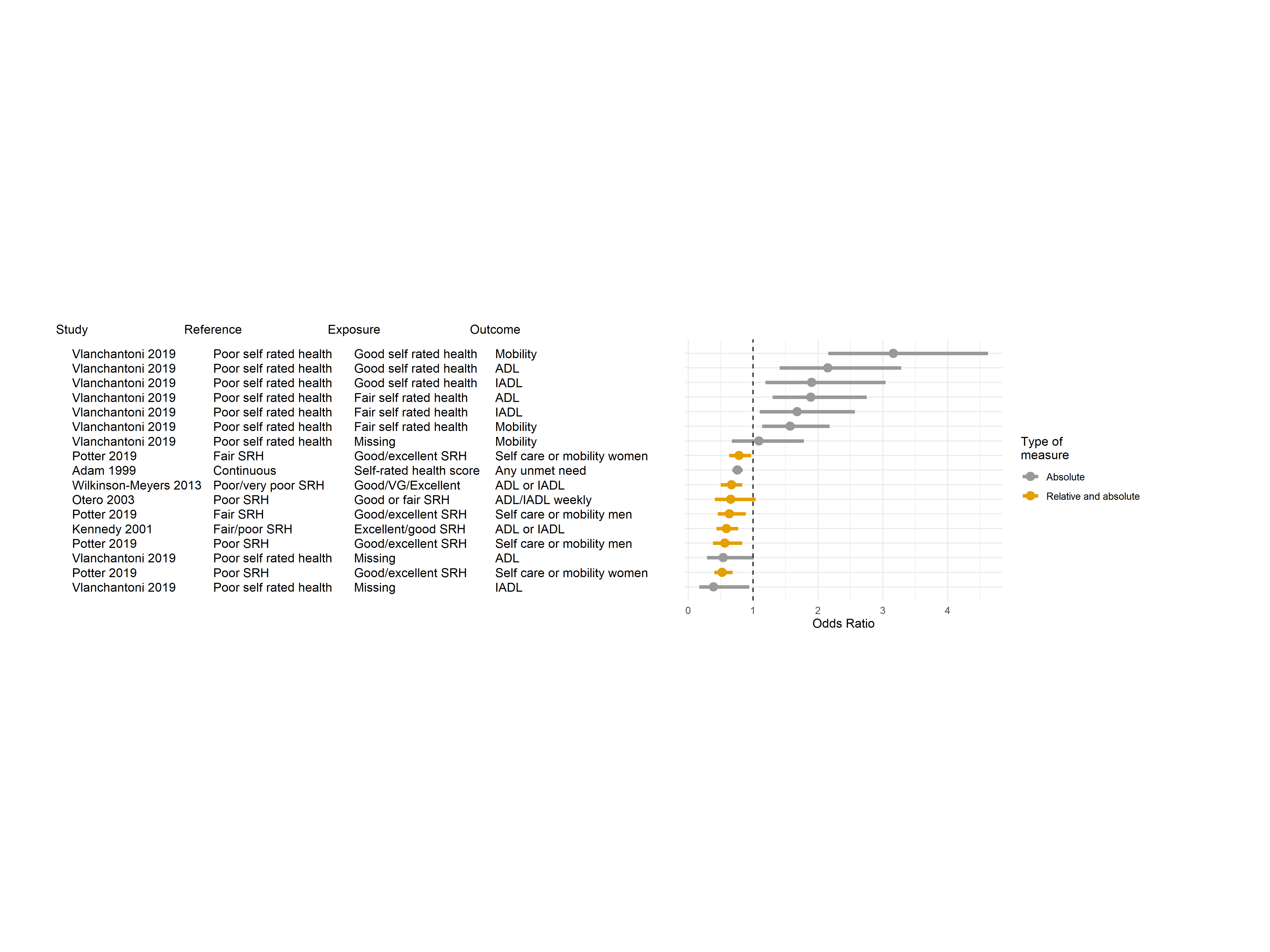


Figure 4b. Presence of functional difficulties and odds of unmet need


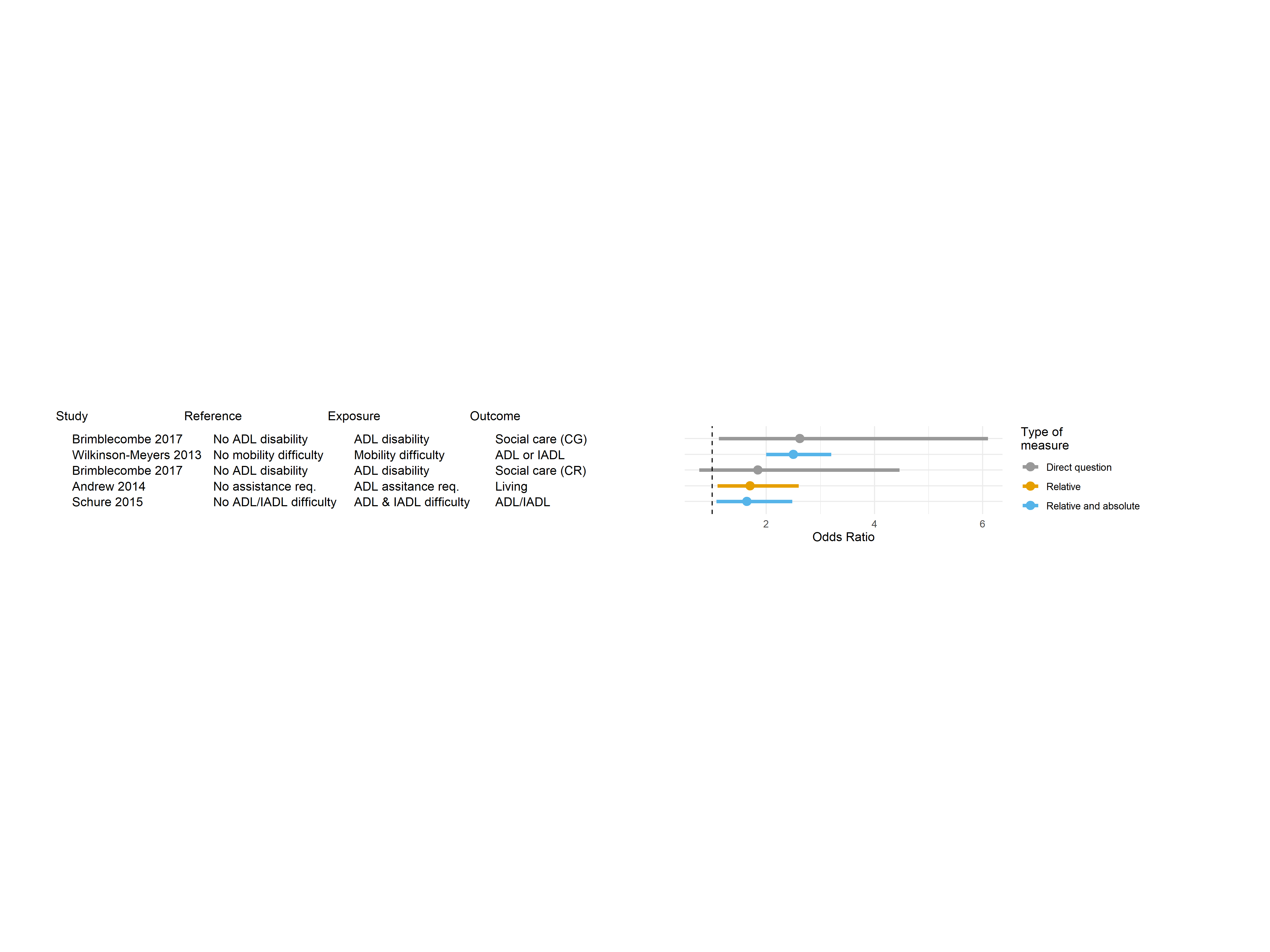


Figure 4c. Number or volume of functional difficulties and odds of unmet need


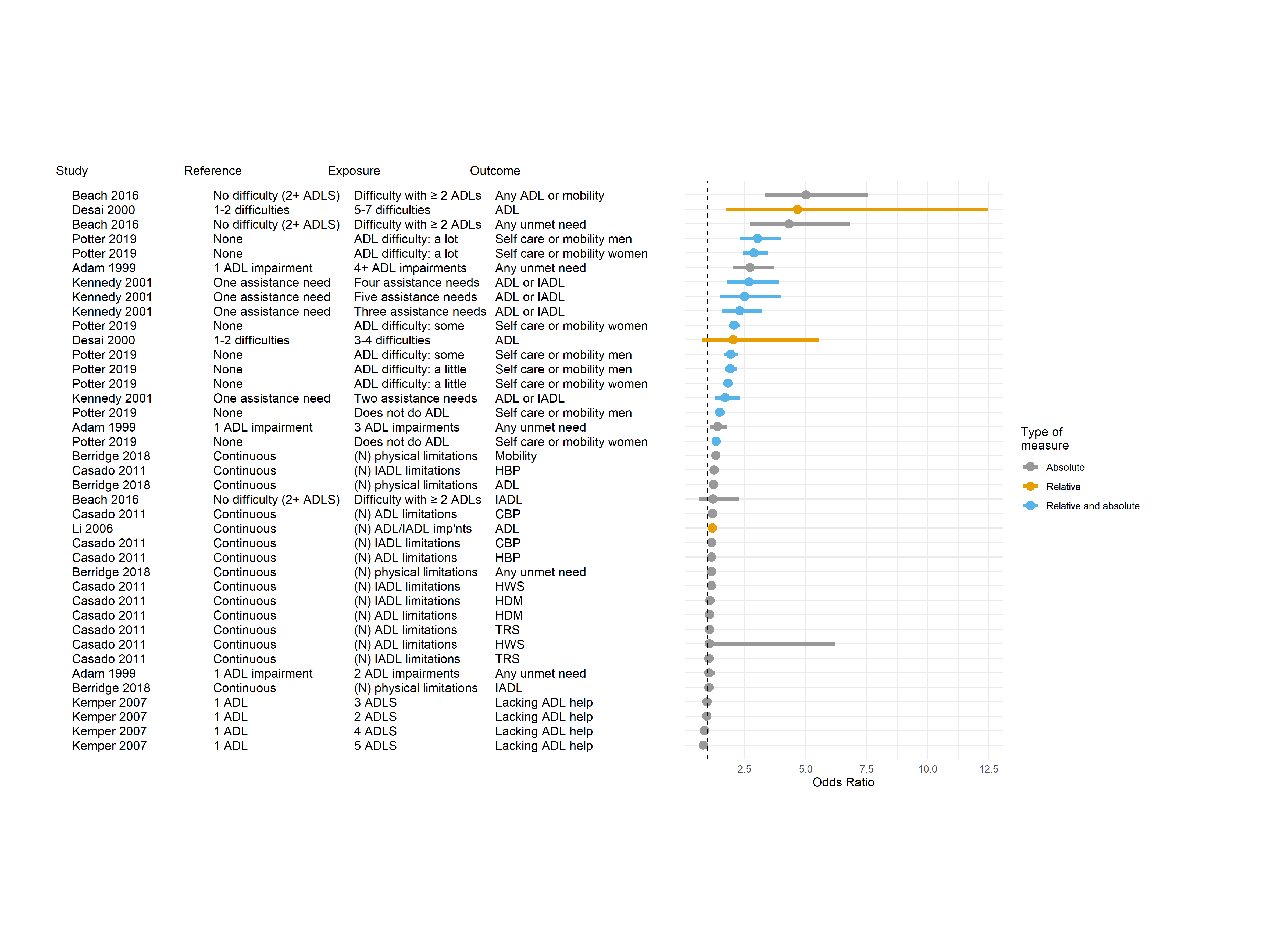


Figure 4d. Scores of physical functioning and odds of unmet need^a,b^


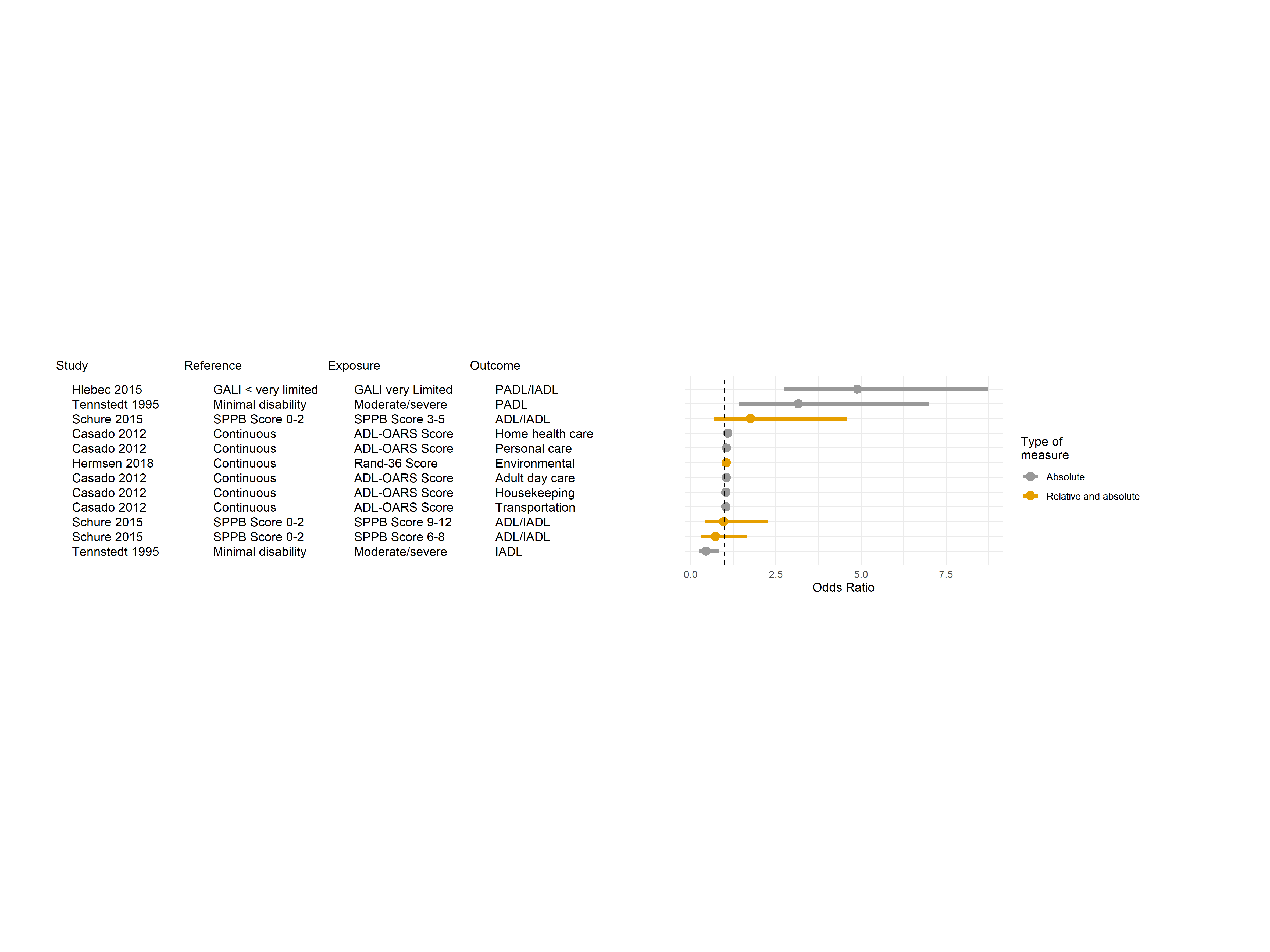


^a^ Different measures of physical functioning were used across studies, therefore unit change is not comparable; ^b^ Higher scores = poorer physical functioning (except Rand SF-36, which has been inverted for this plot)

Figure 4e. Number of health conditions and odds of unmet need


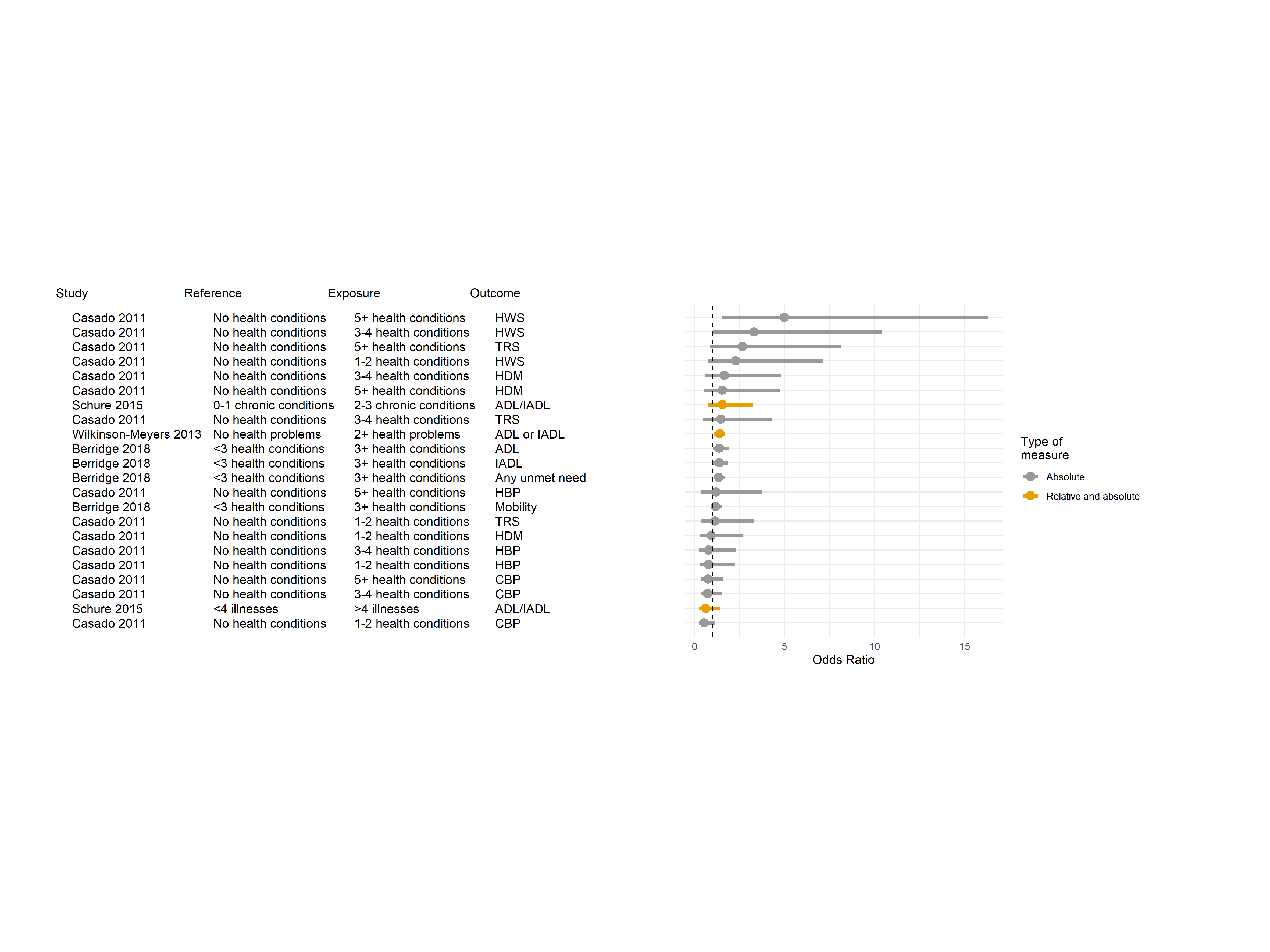


Figure 4f: Presence of a limiting illness and odds of unmet need


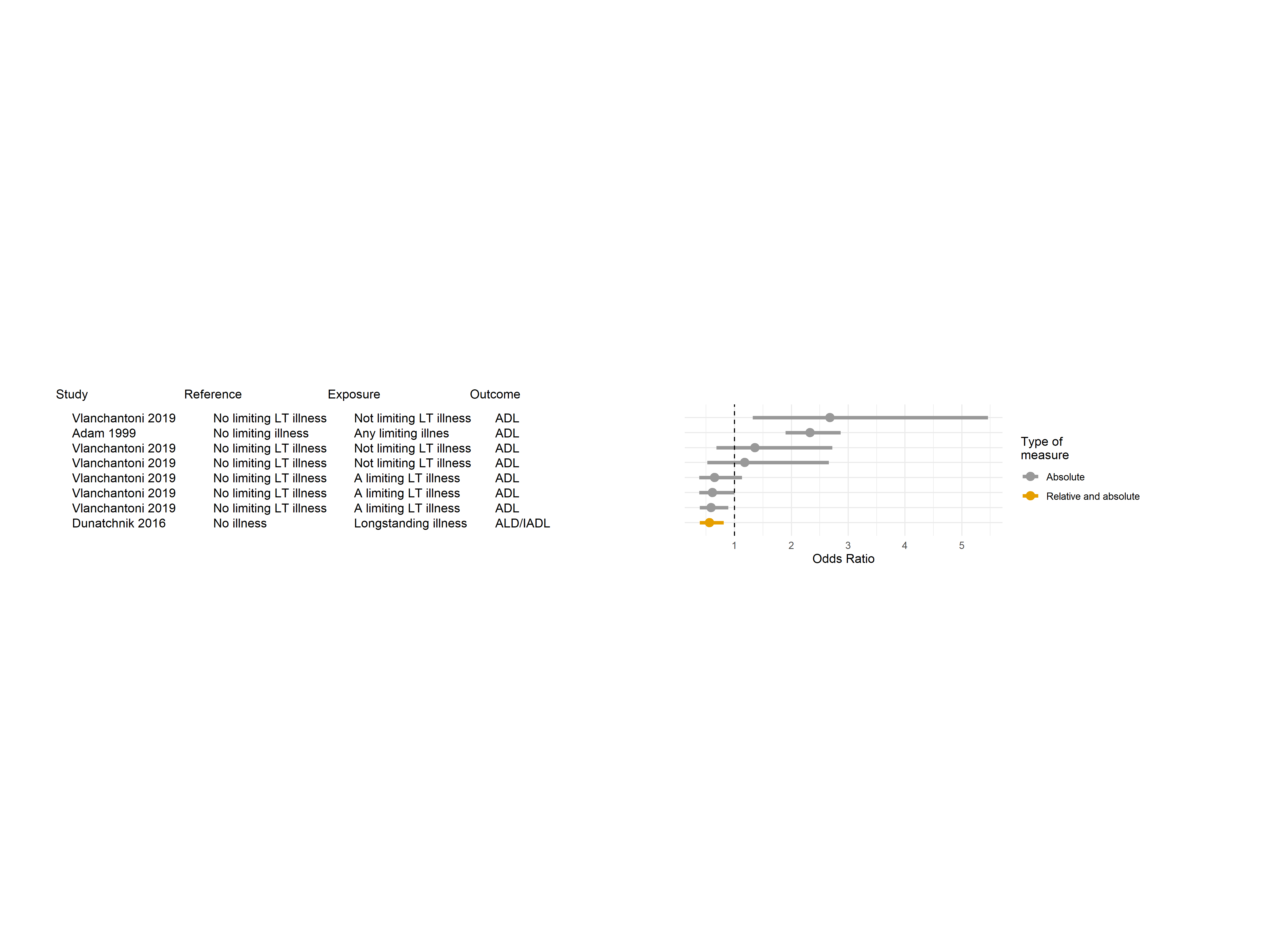


Figure 4g. Arthritis and odds of unmet need


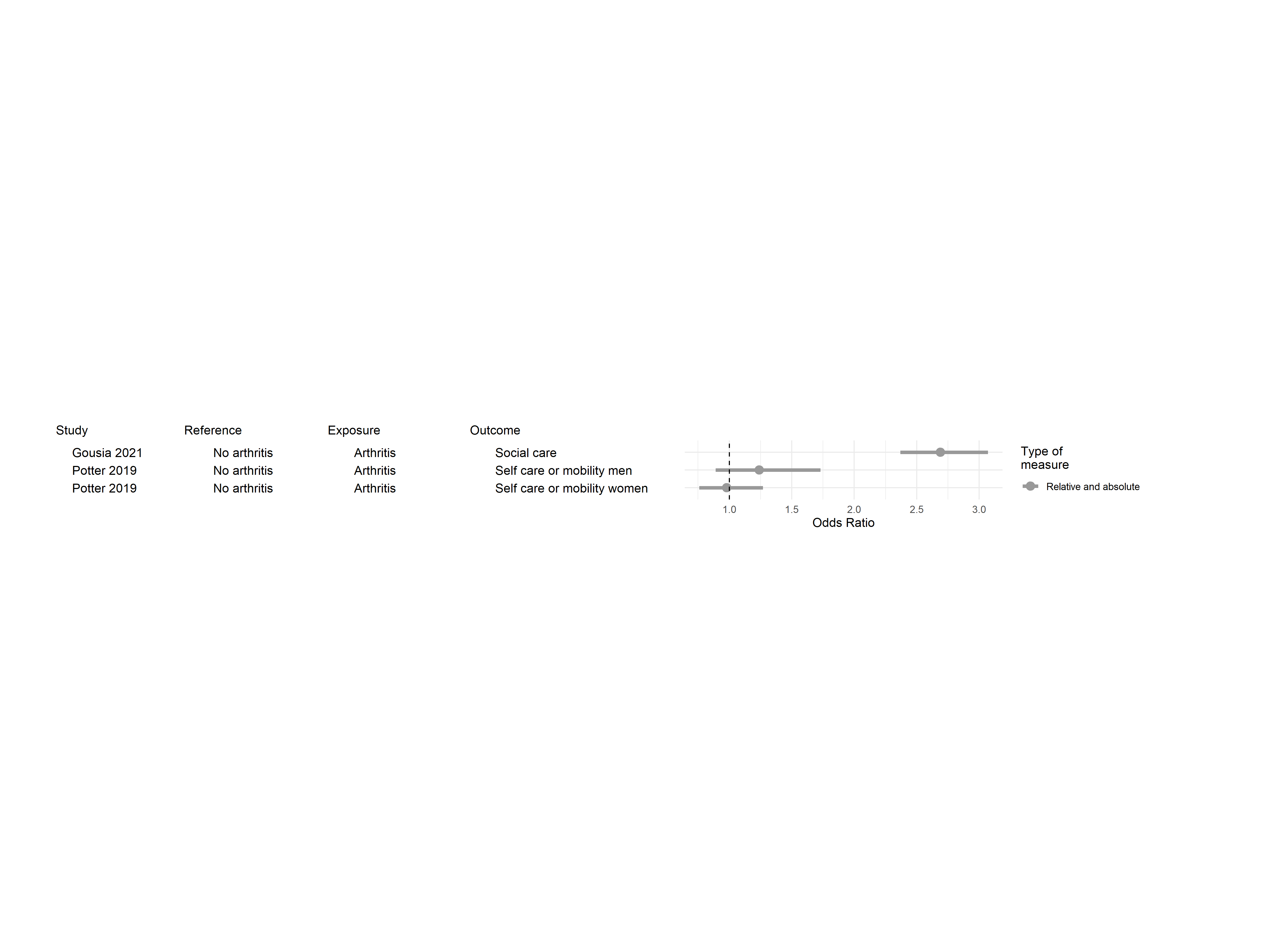


Figure 4g. Cancer and odds of unmet need


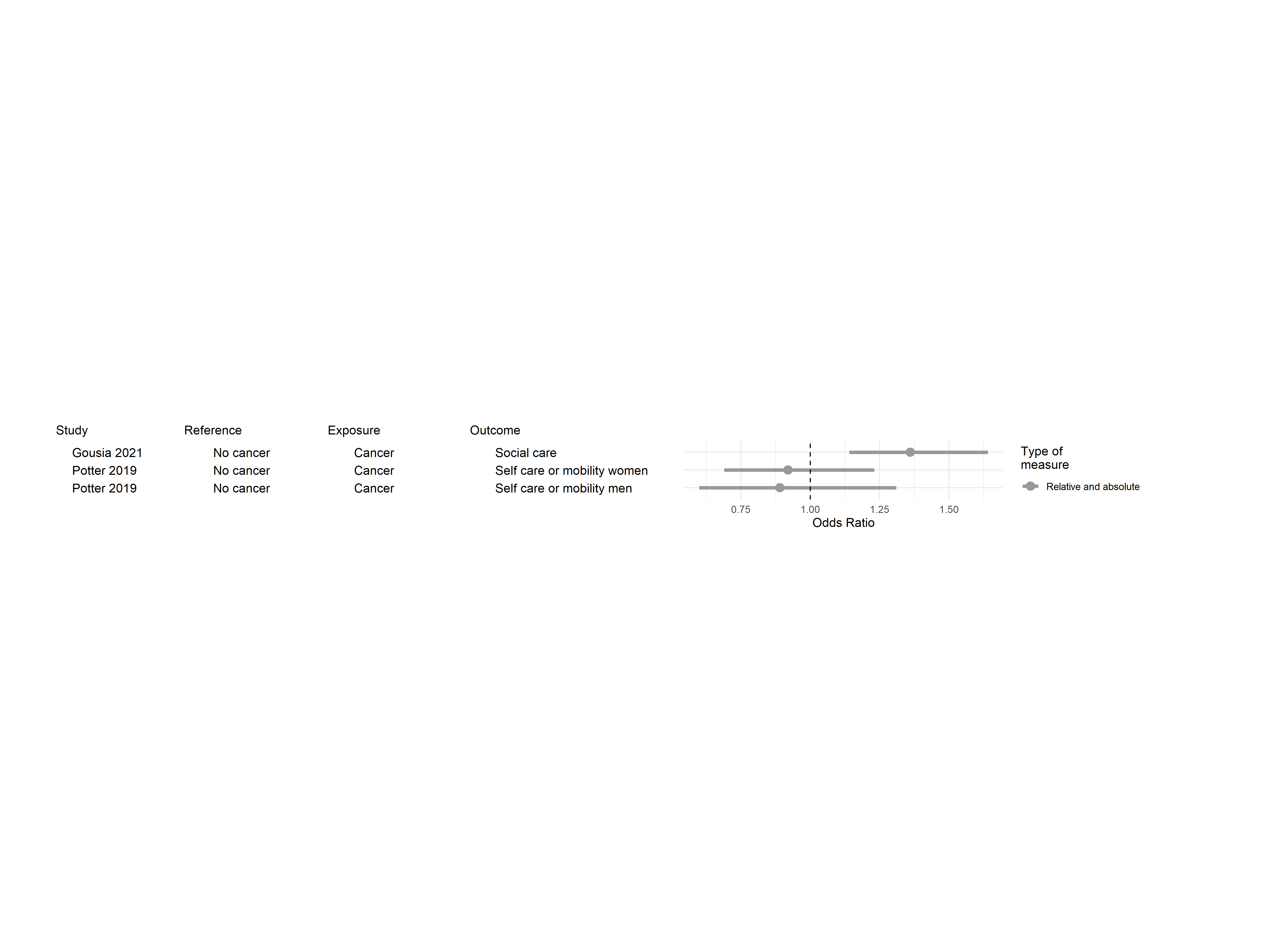


Figure 4h. Cognitive impairment and odds of unmet need


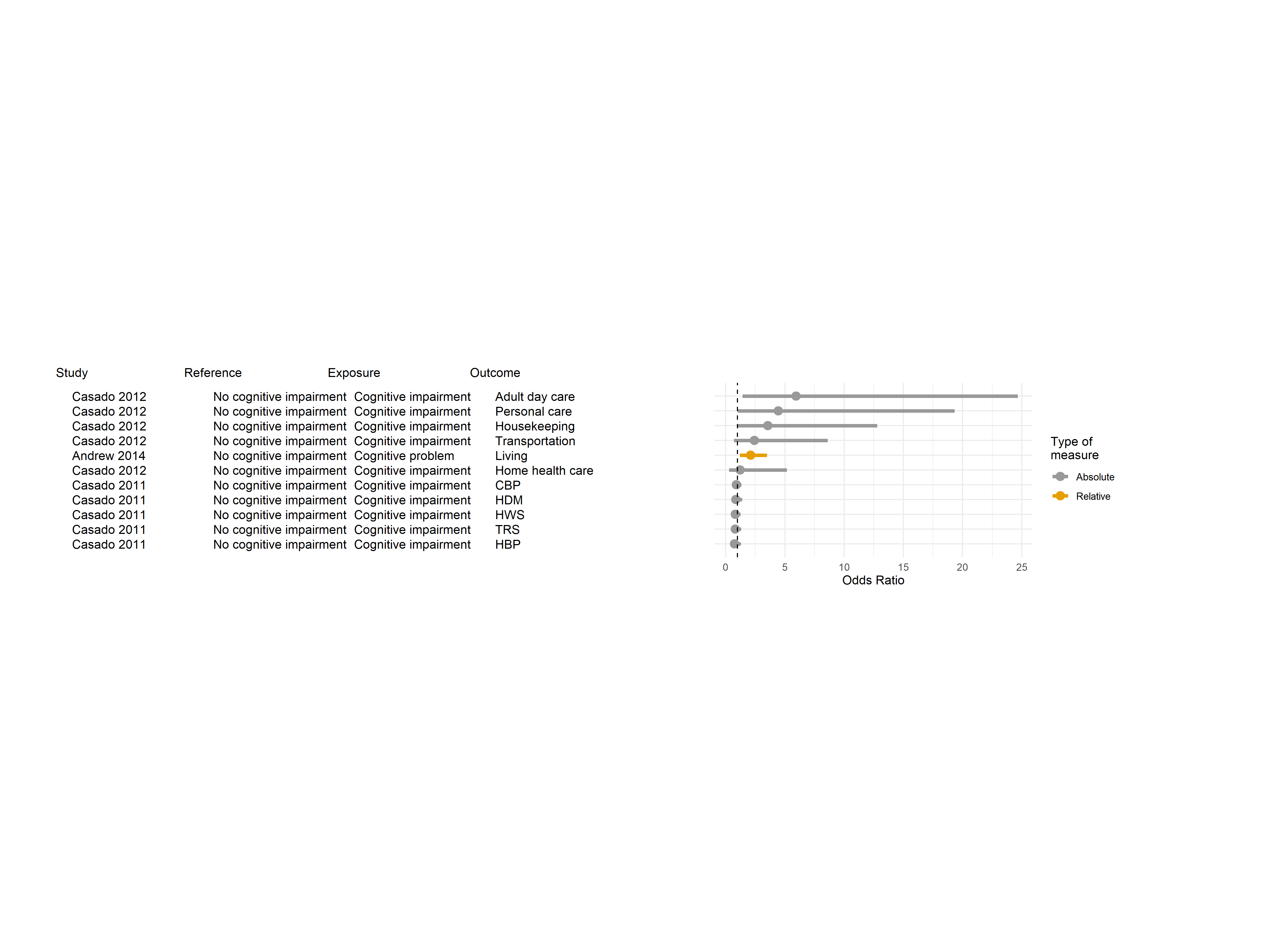


Figure 4i. Dementia and odds of unmet need


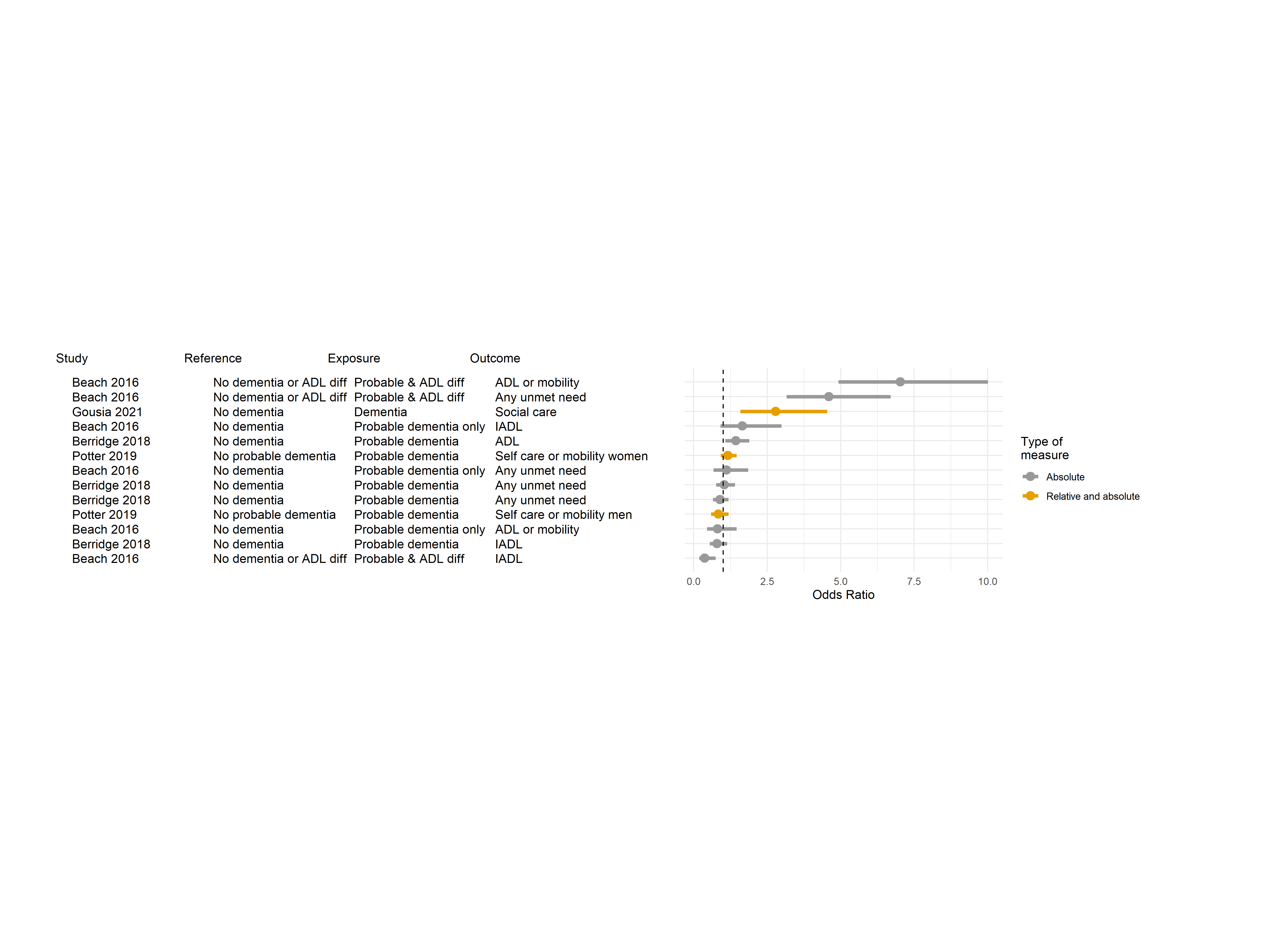


Figure 4j. Depression and odds of unmet need


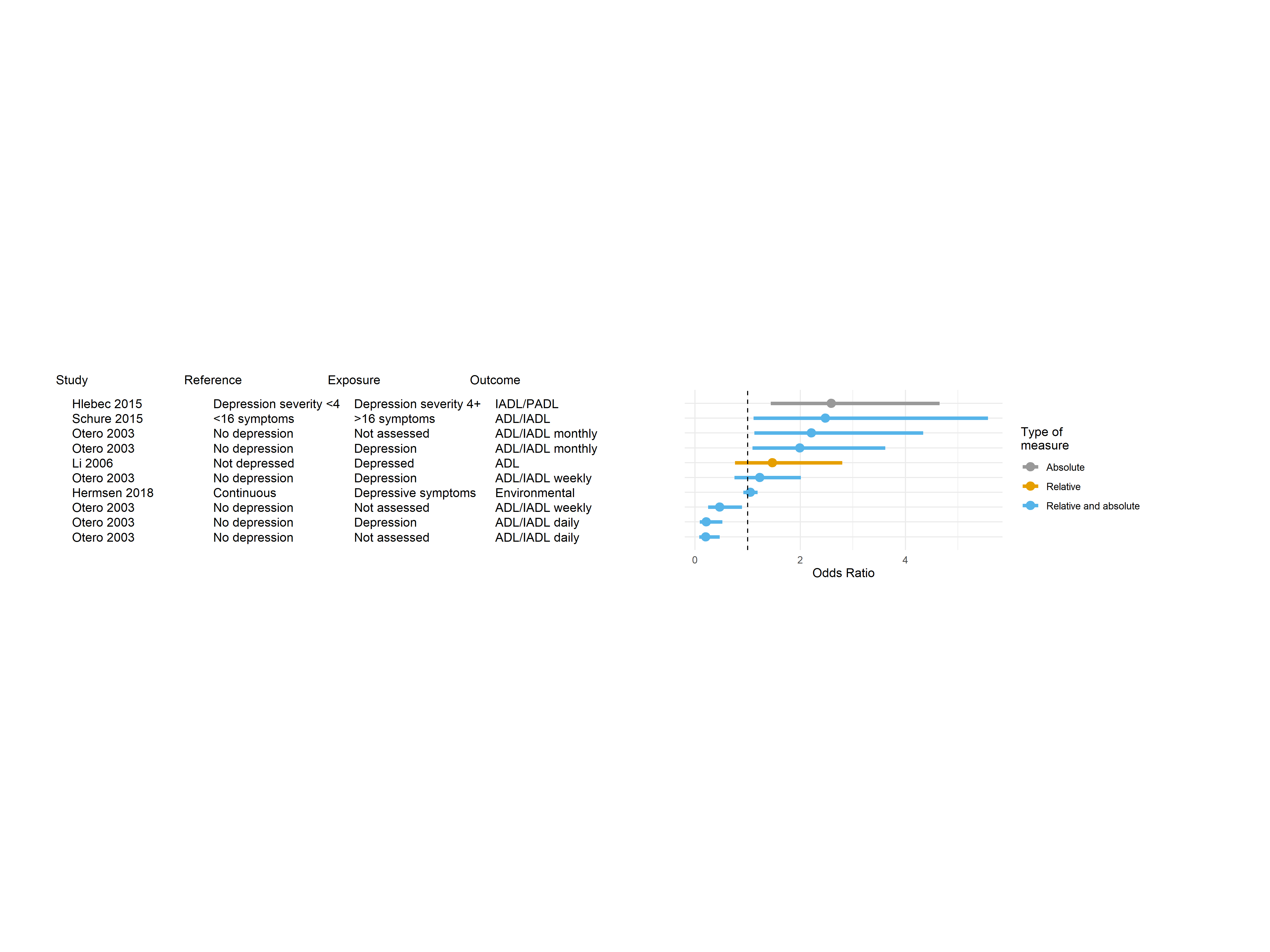


Figure 4k. Diabetes and odds of unmet need


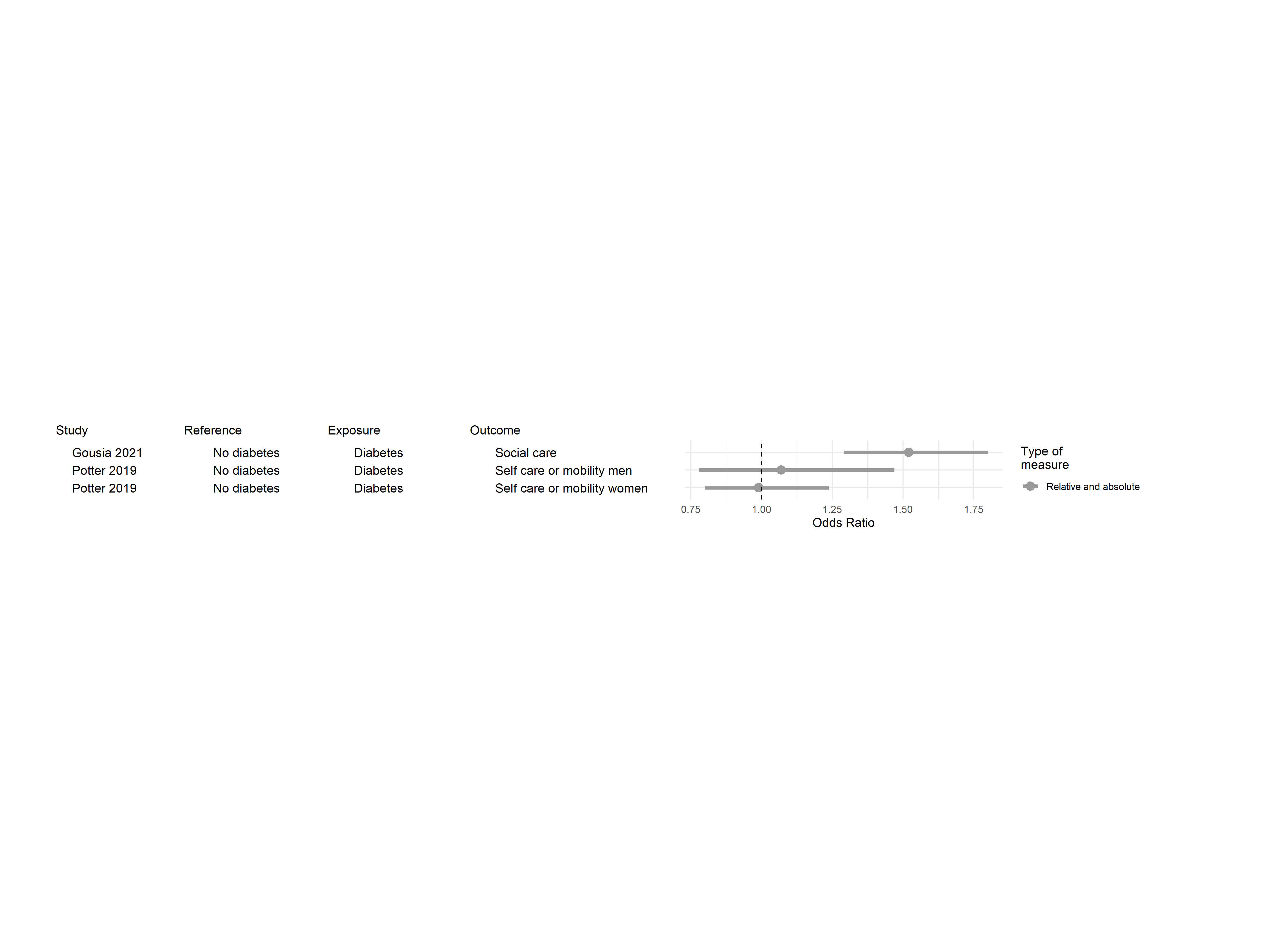


Figure 4l. High blood pressure and odds of unmet need


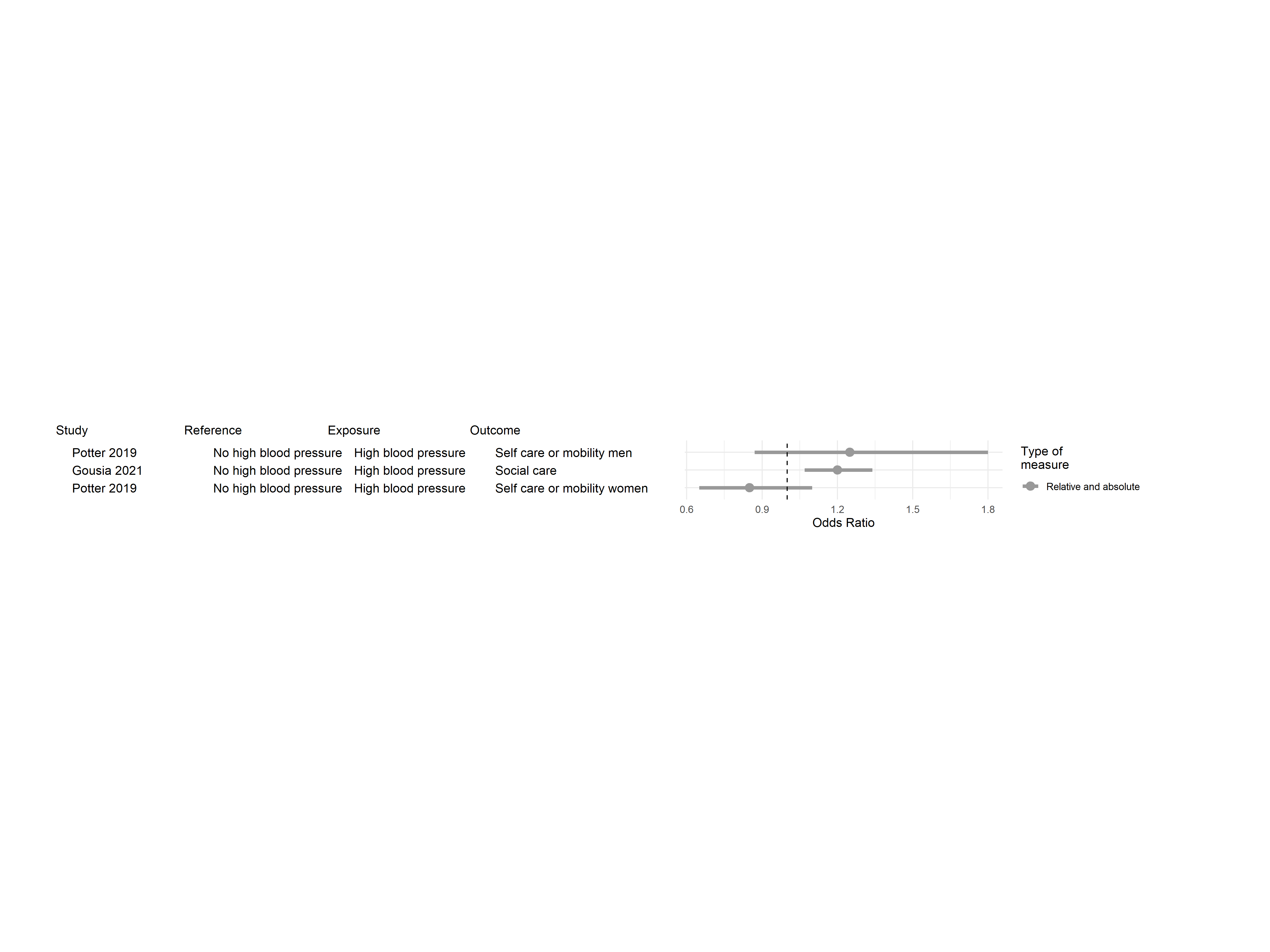


Figure 4n. Osteoporosis and odds of unmet need


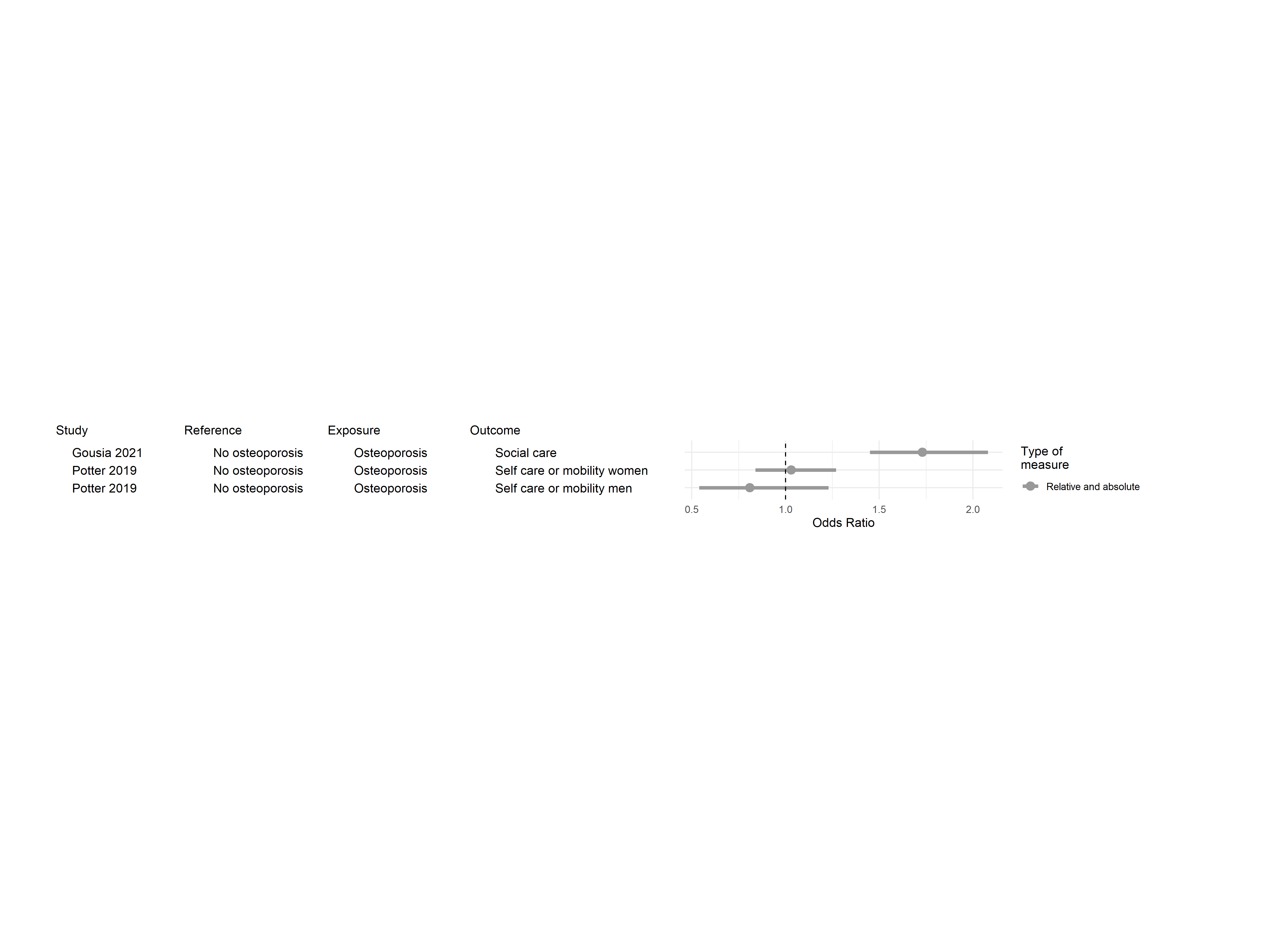


Figure 4o. Stroke and odds of unmet need


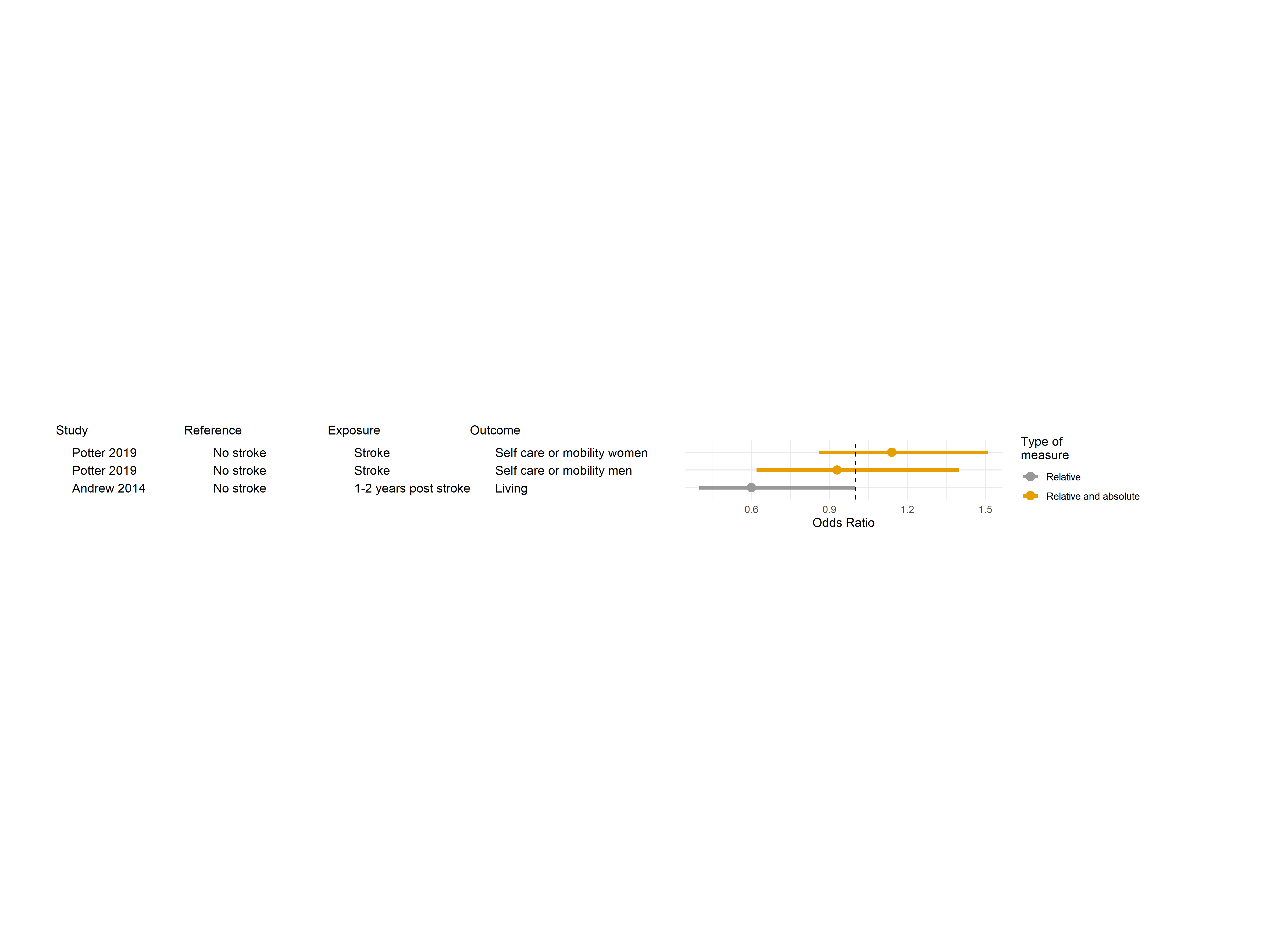


Figure 5a. Carers’ age and odds of unmet need


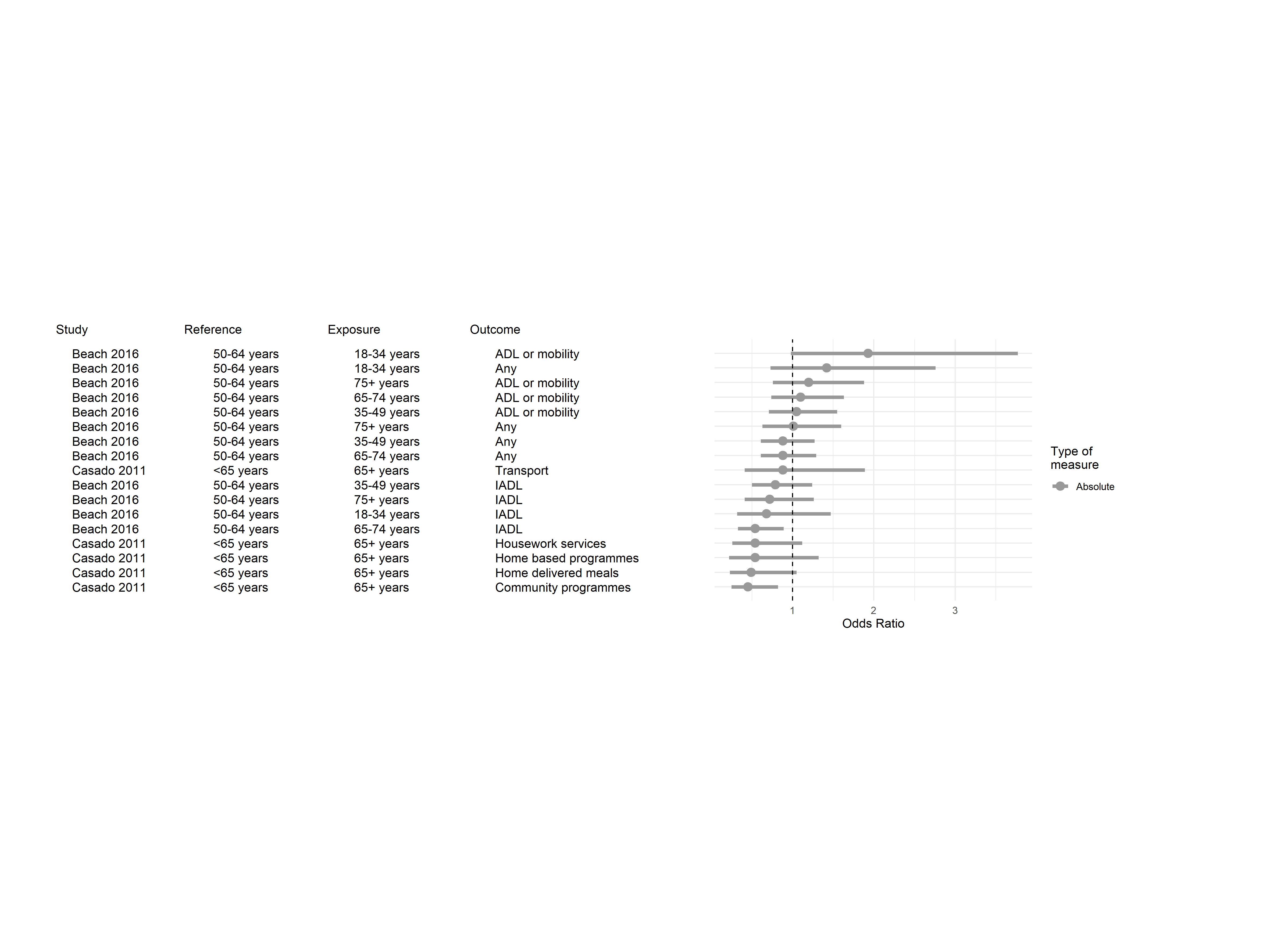


Figure 5b. Carer sex and odds of unmet need


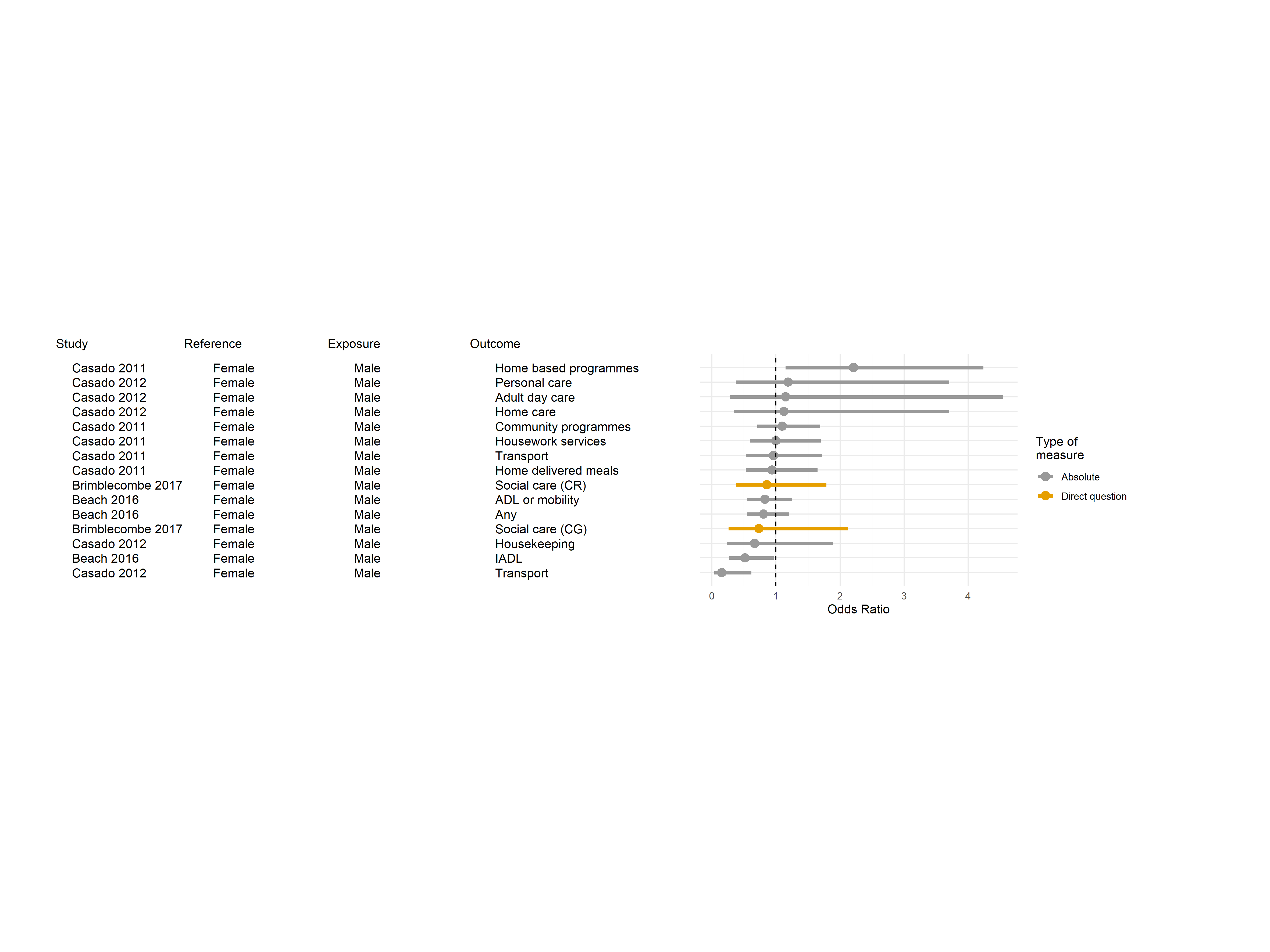


Figure 5c. Cares’ educational attainment and odds of unmet need


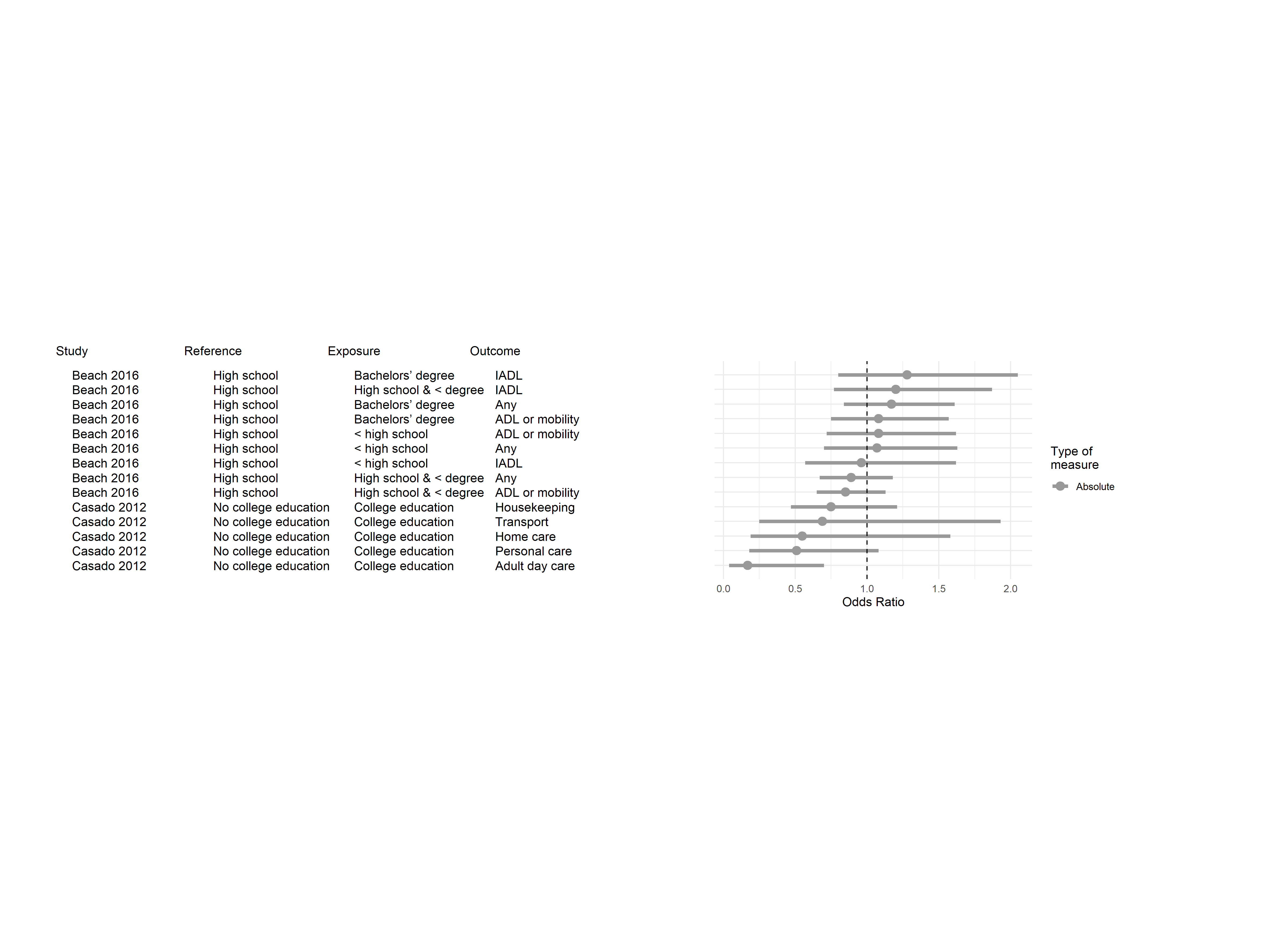


Figure 5d. Carers’ self-rated health and odds of unmet need


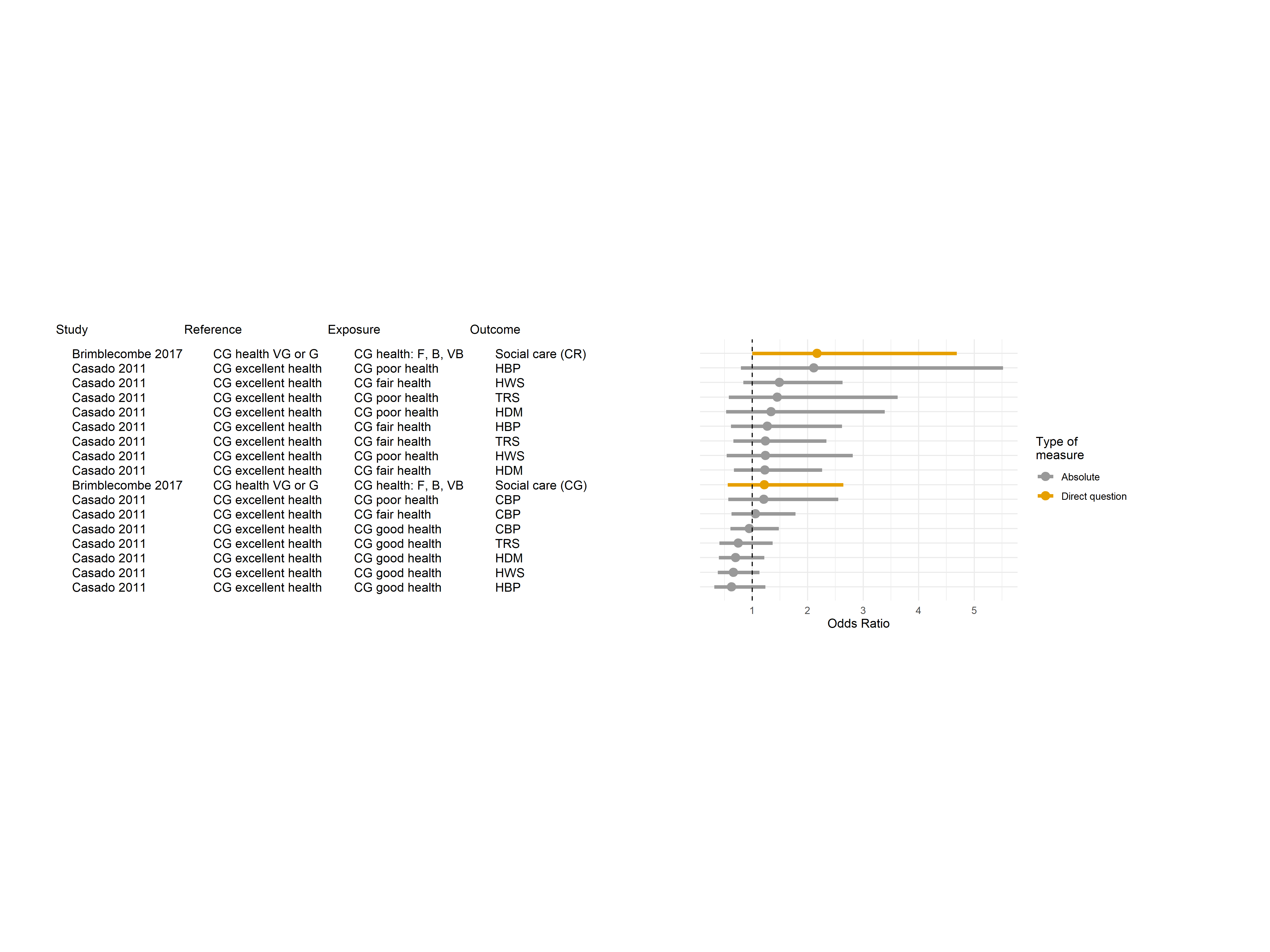

Supplement: aa-22-0904-File002_afac228 [file aa-22-0904-file002_afac228.zip › aa-22-0904-File002_afac228.docx]
